# Phage-encoded factor stimulates DNA degradation by the Hna anti-phage defense system

**DOI:** 10.1038/s41467-026-73157-2

**Published:** 2026-05-18

**Authors:** Matthew M. Hooper, Benjamin T. Hoover, Hongshan Zhang, Adam S. Franco, Ilya J. Finkelstein, David W. Taylor

**Affiliations:** 1 Department of Molecular Biosciences, University of Texas at Austin, Austin, TX USA; 2 Interdisciplinary Life Sciences Graduate Programs, Austin, TX USA; 3 Center for Systems and Synthetic Biology, University of Texas at Austin, Austin, TX USA; 4 LIVESTRONG Cancer Institutes, Dell Medical School, Austin, TX USA

**Keywords:** Cryoelectron microscopy, Enzyme mechanisms, Bacteriophages, Bacterial structural biology, Molecular biology

## Abstract

Prokaryotic organisms have evolved unique strategies to acquire immunity against the constant threat of bacteriophage (phage) and mobile genetic elements. Hna is a broadly distributed anti-phage immune system that confers resistance against diverse phage by eliciting an abortive infection response. Using a combination of biochemistry, cryo-electron microscopy, and single-molecule fluorescence imaging, we reveal that Hna functions as a 3’—5’ single-stranded DNA exonuclease that forms an auto-inhibited dimer under physiological ATP concentrations. Biochemical and mutational analyses demonstrate that Hna catalytic outputs are governed by kinetic partitioning between ATPase and nuclease active sites. Disruption of this balance enhances DNA cleavage and causes cellular toxicity. Furthermore, we show that a phage-encoded single-stranded DNA-binding protein (5 A SSB) destabilizes the autoinhibited Hna dimer and shifts catalytic partitioning toward dysregulated nuclease activation. Conversely, phage escape mutants encode SSB variants that evade Hna surveillance by adopting higher order stoichiometries with enhanced DNA binding affinity. Our work establishes the molecular basis of Hna-mediated anti-phage activity and provides insights into how phage-encoded proteins can directly stimulate a bacterial immune response.

## Abstract (web summary)

Bacteria use diverse immune systems to defend against viral infection. Here, Hooper et al. show that the Hna system is an ATP-regulated DNA nuclease activated by phage proteins. They further reveal how viruses can both trigger and evade this bacterial immune response.

## Introduction

Prokaryotic life is continually challenged by parasitic genetic elements, such as bacteriophage (phage), that contribute disproportionately to bacterial death and are ubiquitous to every environment in which bacteria can grow^1,2^. The environmental pressures imposed by phage have driven bacteria to evolve a diverse repertoire of resistance strategies to neutralize or limit the spread of infection^3–6^. Many well-characterized anti-phage systems, such as CRISPR-Cas and restriction modification (RM) systems, employ targeted nucleic acid degradation to disrupt replication of the phage genome^5,7,8^. By contrast, a distinct subclass of bacterial immune systems, known as abortive infection (abi) systems, often interfere with viral propagation by weaponizing host cellular processes and triggering cell death or dormancy to benefit the surrounding bacterial population^9–11^. Recent studies highlight the mechanistically diverse and underexplored strategies of abi systems, emphasizing the potential for the discovery of novel approaches used by bacteria to combat phage^12–16^.

Hna (helicase-nuclease abortive infection) is an abi defense system originally discovered in the gram-negative bacterium, *Sinorhizobium meliloti*, that has been identified in ~2% of sequenced bacterial genomes^15,17–19^. Foundational work by Sather et al. established that Hna functions as a single-effector system to confer immunity against diverse families of tailed viruses (*Caudoviricetes*). Hna interferes with phage genome replication without releasing phage progeny via an unknown mechanism^15^. Hna possesses highly conserved N-terminal superfamily II (SF2) helicase motifs and a C-terminal PD-(D/E)XK nuclease domain. The cooperativity of nuclease and helicase modules is an established approach for defense against phage attack and has been characterized in functionally distinct systems such as Hachiman, Gabija, Nhi, and Cas3^14,20–25^. Conservation of both nuclease and helicase modules is often necessary for proper anti-phage activity, however, the interplay of each domain is highly variable across systems, allowing for unique modes of activation and regulation.

Recently, studies have identified several phage-encoded protein factors that induce abortive infection through direct or indirect activation of bacterial immune systems^26–29^. Of growing interest are phage-encoded single-stranded DNA binding proteins (SSB) which have been shown to stimulate the anti-phage activity of several defense systems including Hachiman, Nhi, Retron-Eco8, AbpAB, and Hna^15,24,30,31^. Co-expression of a phage-encoded SSB is sufficient to induce Hna-mediated abi independent of phage infection^15^ and establishes a unique opportunity to explore the nature of Hna anti-phage activity and the external factors that regulate it.

Here, we characterize the catalytic properties of the Hna system originating from *S. meliloti*. We demonstrate that Hna functions as a 3’—5’ exonuclease on single-stranded DNA and forms an auto-inhibitory homodimer upon binding ATP. Disrupting either the dimerization or ATP-binding domains restores Hna exonuclease activity, establishing a mode of self-regulation under normal cellular conditions. Addition of a phage-encoded SSB (5 A SSB) stimulates Hna exonuclease activity even in the presence of ATP, disrupting Hna auto-inhibition. Together, this work details the mechanism of Hna broad-spectrum anti-phage activity and contributes to our understanding of phage-encoded triggers for activation of abortive infection systems.

## Results

### Hna is a single-stranded DNA exonuclease

**Fig. 1:**
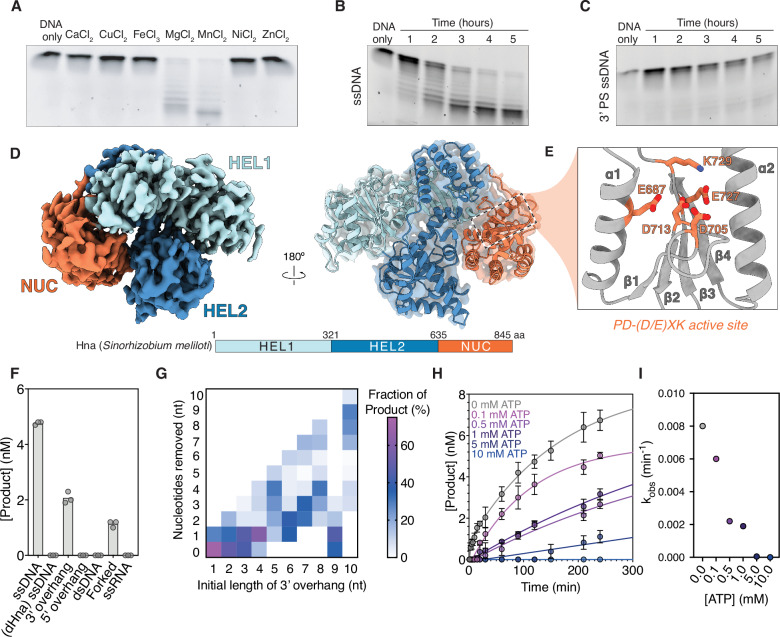
Hna is a 3’—5’ single-stranded DNA exonuclease that is negatively regulated by ATP. **A** Hna exonuclease activity using fluorescently labeled, single-stranded DNA oligonucleotide in the presence of various metal ions and absence of ATP. Gel shown is representative of 4 independent experiments. **B** Exonuclease activity over time using ssDNA without or **C** with a series of 3’-phosphorothioate (PS) modifications. Gels shown are representative of 3 independent experiments. **D** Cryo-EM structure of monomeric Hna at a resolution of 3.9 Å with Hna domain organization shown below (HEL1, Superfamily 2 helicase 1 in light blue; HEL2, Superfamily 2 helicase 2 in dark blue; NUC, PD-(D/E)XK nuclease in orange). **E** Magnified view of PD-(D/E)XK nuclease active site showing significant catalytic residues and typical secondary structure of PD-(D/E)XK nuclease active sites. **F** Quantification of DNA cleavage by Hna in the presence of various substrates using capillary electrophoresis. Individual data points shown are from three biological replicates Data shown are three independent reactions incubated at 37 °C for 1 hour. **G** Quantification of DNA degradation by Hna using substrates containing varying length of 3’ ssDNA overhangs. The length of each product formed was measured using capillary electrophoresis to determine the number of nucleotides removed. **H** Quantification of ssDNA cleavage by Hna in the presence of varying concentrations of ATP using capillary electrophoresis. Data shown are the mean ± standard deviation of three independent experiments for each condition, fit to a single-exponential equation. **I**, Rates of ssDNA cleavage across varying ATP concentrations, derived from fit of three independent experiments. Source data are provided as a Source Data file.

To investigate the putative nuclease capabilities of Hna, we recombinantly expressed and purified the Hna gene originating from *Sinorhizobium meliloti* in *Escherichia coli*. We first evaluated the ability of Hna to degrade single-stranded DNA (ssDNA) in the presence of various metal ions. Hna exhibited Mg^2+^ and Mn^2+^-dependent ssDNA degradation with no discernible nuclease activity in the presence of Ca^2+^, Cu^2+^, Fe^3+^, Ni^2+^, or Zn^2+^ (Fig. 1A). To determine the relative efficiency of Hna nuclease activity, we assessed ssDNA degradation over time and observed slow but continuous cleavage over extended time scales (Fig. 1B). Additionally, to probe the directionality of nuclease activity, we introduced nuclease-resistant phosphorothioate (PS) modifications at either the 3’ or 5’ end of the ssDNA. Hna was unable to degrade ssDNA with 3’ PS modifications but was unperturbed by the inclusion of 5’ PS modifications, indicating that Hna functions as a 3’—5’ exonuclease (Fig. 1C, Supplementary Fig. 1A, 1B).

We next aimed to elucidate the molecular mechanism underlying Hna-mediated nuclease activity. We used cryo-electron microscopy (cryo-EM) to determine a structure of Hna at a resolution of 3.9 Å by combining Hna with 3’-PS ssDNA and the non-cleavage competent metal, Ca^2+^ (Fig. 1D). Hna is an 845-amino acid protein with two, N-terminal SF2 helicase domains (HEL1, HEL2) and a C-terminal PD-(D/E)XK nuclease-like domain (NUC)^32,33^. Structural analysis revealed that Hna resembles a subclass of DinG-like proteins, termed ExoDinG, that also function as 3’—5’ ssDNA exonucleases^34,35^. Comparison of Hna with structurally similar members of the DinG and XPD protein families revealed that Hna retains characteristic sequence motifs associated with ATP binding and hydrolysis in SF2 proteins, as well as a reduced Arch domain, yet lacks the canonical iron-sulfur cluster (Supplementary Fig. 2A-C)^32,34,36–39^.

The C-terminal NUC domain of Hna contains a highly-conserved, catalytic core associated with PD-(D/E)XK nucleases (αβββαβ configuration) with minimal structural conservation in the surrounding scaffold (Fig. 1E)^33,40^. We mutated a single residue, K729A, in the nuclease active site to develop a nuclease-dead Hna mutant (*dHna*). Substitution of this residue was sufficient to completely abrogate Hna exonuclease activity (Fig. 1F). In addition to ssDNA, we also assessed whether Hna could degrade double-stranded DNA (dsDNA), partially duplexed DNA, or ssRNA. Using high-resolution capillary electrophoresis (CE), we observed that Hna degrades all DNA substrates that contain single-stranded 3’ ends and exhibits no nuclease activity with ssRNA, dsDNA, or 5’ ssDNA overhangs (Fig. 1F, Supplementary Fig. 1B).

Given the observation that Hna can degrade DNA overhangs, we performed additional cleavage experiments by incubating Hna with substrates containing varying lengths of 3’ ssDNA overhangs. Using CE, we can accurately measure the length and distribution of cleavage products produced by Hna for each substrate. We observed that Hna cleavage products converge on lengths consistent with the total length of the ssDNA overhangs, suggesting that Hna likely degrades ssDNA processively but is unable to bypass double-stranded DNA junctions (Fig. 1G, Supplementary Fig. 1C). Unexpectedly, Hna exhibited relatively low levels of nuclease activity when presented with a substrate containing a 9-nt ssDNA overhang. In the absence of a DNA-bound structure, we leveraged AlphaFold3 (AF3)^41^ structural prediction to visualize Hna bound to a 9-nt ssDNA substrate (Supplementary Fig. 2D). Inspection of the AF3 model shows that the 9-nt substrate fully occupies the DNA binding site, likely recapitulating the DNA footprint of Hna. Additionally, overlay of the AF3 model with our determined Hna structure revealed a unique conformation of the NUC domain when bound to the 9-nt ssDNA that appears largely disengaged from the HEL domains as observed in our Hna monomer structure. We speculate that the precise occupancy of the DNA binding domain results in non-productive rearrangements of the NUC domain, contributing to the notable loss of nuclease activity.

### ATP inhibits Hna nuclease activity

Due to the highly conserved ATP-binding motifs, we questioned whether the inclusion of ATP could modulate Hna nuclease activity. To assess this we performed a series of time-course ssDNA cleavage experiments over a range of ATP concentrations (0 mM-10 mM) and observed significant reduction in ssDNA cleavage across all concentrations of ATP tested (Figs.1H, 1I). Additionally, substitution of ATP for the non-hydrolyzable analog, ADP·BeF_3_, yielded comparable inhibition of DNA degradation, suggesting that Hna enters an inhibited state upon ATP binding rather than following ATP hydrolysis (Supplementary Fig. 3C)^42–44^. These data suggest that ATP acts as a negative regulator of Hna nuclease activity with considerable sensitivity at even sub-physiological levels of ATP ( < 1 mM)^45,46^.

**Fig. 2:**
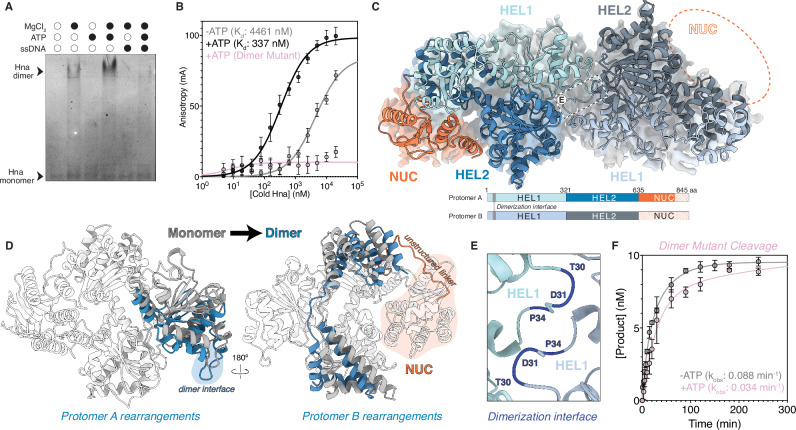
Hna forms an auto-inhibited dimer. **A** Native gel shift assay to assess the basis of Hna dimerization. All reactions were incubated for 1 hour at 37 °C then evaluated using non-denaturing gel electrophoresis. Gel shown is representative of three independent experiments. **B** Fluorescence anisotropy of GFP–Hna (fixed concentration) was monitored during titration with increasing concentrations of unlabeled (cold) wild-type (gray and black) or dimer mutant (pink) Hna in the absence or presence of ATP. Data points represent mean ± SD from three biological replicates and were fit to a one site specific binding model to derive apparent dissociation constants **C** Cryo-EM structure of dimeric Hna at a resolution of 4.4 Å with domain organization of each protomer shown below (HEL1, Superfamily 2 helicase 1 in light blue; HEL2, Superfamily 2 helicase 2 in dark blue; NUC, PD-(D/E)XK nuclease in orange). Dimerization interface highlighted in gray and unresolved portions of each nuclease domain represented by a crosshatched pattern. **D** Domain rearrangements observed in protomer A or protomer B upon Hna dimerization. Structure of Hna dimer (blue) overlaid with structure of Hna monomer (gray). **E** Magnified view of Hna dimer interface denoting key residues in unstructured HEL1 loop (T30, D31, and P34). **F** Quantification of DNA cleavage by Hna dimer mutant (T30A/D31A/P34A) in the presence of ssDNA with or without the addition of ATP using capillary electrophoresis. Data shown are the mean ± standard deviation of three independent experiments for each condition, fit to a double-exponential equation. Source data are provided as a Source Data file.

To understand the molecular mechanism of ATP-mediated nuclease inhibition, we performed a native gel shift assay after incubating Hna with a combination of ssDNA and various co-factors including Mg^2+^ and ATP. We observed that Hna stochastically forms a higher-order complex that is greatly enriched in the presence of ATP (Fig. 2A). To more accurately probe the nature of Hna oligomerization, we performed a series of fluorescence anisotropy experiments using Hna with a C-terminal GFP tag. Holding GFP-Hna concentration constant, we titrated the concentration of unlabeled Hna and monitored changes in anisotropy in the absence or presence of ATP. The resulting binding curves revealed that ATP stimulates a ~ 13-fold increase in Hna oligomerization (Fig. 2B).

Characteristic of SF2 helicase proteins is their ability to form stable homodimers, often with the dimeric state conferring increased activity^32,47^. For Hna, however, we postulated that ATP-binding facilitates the formation of an Hna homodimer with reduced nuclease activity. To elucidate the molecular mechanism of Hna autoinhibition, we determined a cryo-EM structure of an Hna dimer in the presence of ADP·BeF_3_ and 3’-PS ssDNA at a global resolution of 4.4 Å (Fig. 2C). Despite limitations in the final resolution of this structure, we were able to rigid-body fit domains of each Hna protomer to visualize notable domain rearrangements upon Hna dimerization. Hna assembles into a homodimer with psuedo-C2 symmetry via a highly conserved, unstructured loop in the HEL1 domain (Fig. 2C and E). This interaction is stabilized through an asymmetric shift of the HEL1 domain of protomer A, increasing availability of the dimerization interface (Fig. 2D). Critically, we observed that the NUC domain in protomer A was only partially resolved, while fully unresolved in protomer B, indicating a high degree of flexibility in this region (Fig. 2C). Rearrangement of a network of alpha helices in the HEL2 domain of protomer B promotes flexibility of the NUC domain via a long, unstructured linker (Fig. 2D). These asymmetrical movements provide a physical basis for the differential resolvability of the NUC domain in each Hna protomer and, consistent with our AF3 predictions, suggests that NUC flexibility may contribute to decreased nuclease capacity.

To validate the regulatory role of Hna dimer formation, we mutated key residues along the dimerization interface (T30A/D31A/P34A), henceforth referred to as the Hna dimer mutant (Fig. 2E). The dimer mutant failed to stimulate increased anisotropy when titrated in the presence of GFP-Hna, indicating successful disruption of Hna oligomerization (Fig. 2B). We then performed additional time-course ssDNA cleavage experiments using the dimer mutant in the presence of ATP. Strikingly, we observed more than a 10-fold increase in intrinsic Hna cleavage rate compared to wild-type Hna with minimal perturbation upon inclusion of ATP (Fig. 2F). Collectively, these observations indicate that Hna dimer formation, stabilized through ATP binding, substantially inhibits Hna exonuclease activity. Consequently, disruption of the Hna dimer interface leads to enhanced ssDNA exonuclease activity that is minimally regulated by ATP.

### Kinetic partitioning governs Hna catalytic outcomes

**Fig. 3:**
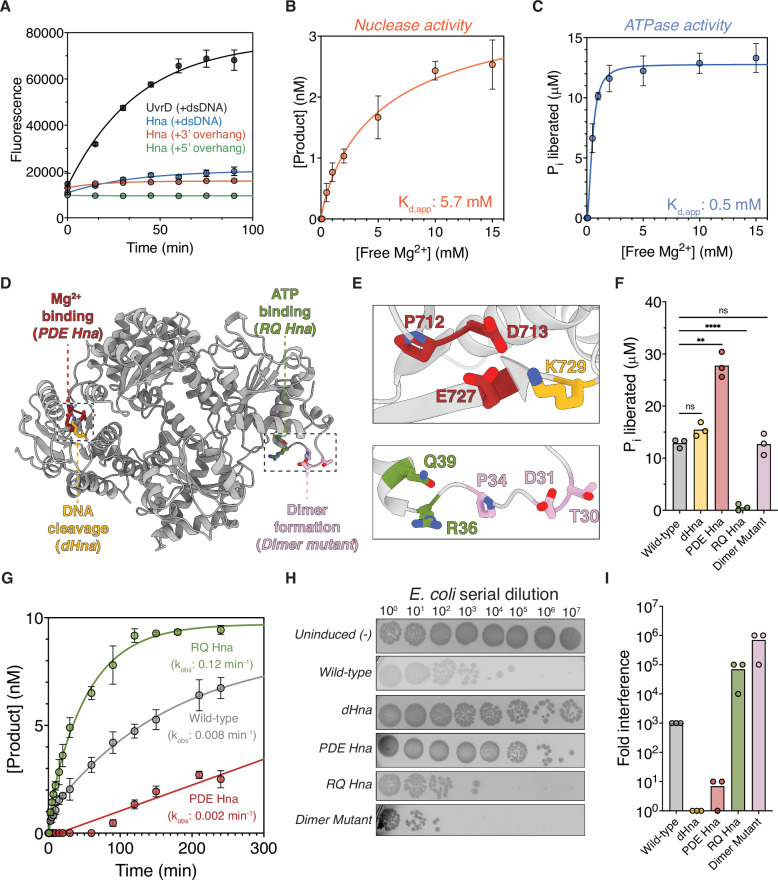
Kinetic partitioning governs Hna catalytic outcomes. **A** DNA unwinding on fully double-stranded or partially duplexed substrates with 15-nucleotide overhangs on either the 3’ or 5’ end of the molecule. At double-stranded ends of each substrate, a 6-FAM label or Black Hole Quencher 1 (BHQ-1) modification is incorporated on either strand to quench fluorescence. All reactions were incubated at 37 °C in the presence of ATP. The commercially available helicase, UvrD, included as a positive control. Data shown are mean ± standard deviation of three independent experiments. **B** Nuclease activity and **C**, ATPase activity measured across a range of free Mg²⁺ concentrations. Plotted values represent raw cleavage product formation or phosphate release ± standard deviation of three biological replicates, quantified after 20 minutes at 37 °C. Data were fit to a single-site binding model to derive apparent Mg²⁺ dissociation constants (K_d,app_) for each activity. **D** Structure of Hna monomer depicting sites of directed mutagenesis **E** Magnified view of sites of mutagenesis with each mutant differentially colored: P712A/D713A/E727A PDE Hna, red; K729A dHna, yellow; R36A/Q39A RQ Hna, green; and T30A/D31A/P34A Dimer Mutant Hna, pink. **F** Malachite green ATPase assay using wild-type Hna or various Hna mutants with single-stranded DNA present. Phosphate concentration was determined reactions after 30 minutes of incubation 37 °C. Individual data points are shown for three biological replicates. Statistical significance is evaluated via two-sided unpaired t tests with p values for each analysis specified in plot; *p  <  0.05, **p  <  0.01, ***p  <  0.001. **G** Quantification of DNA cleavage by Wild-type, RQ, and PDE Hna in the presence of ssDNA using capillary electrophoresis. Data shown are the mean ± standard deviation of three independent experiments for each condition, fit to a single or double-exponential equation. **H** Representative spot assay showing ten-fold serial dilutions of cells expressing the indicated Hna mutants plated under inducing conditions to assess cellular toxicity. Spot assay shown is representative of three independent experiments. **I** Quantification of cellular toxicity assay determined as the fold interference in cell growth compared to uninduced condition. Individual data points are shown for three biological replicates. Source data are provided as a Source Data file.

Next, we sought to characterize the potential DNA unwinding and ATPase capabilities of Hna based on its highly conserved helicase domains. We incubated Hna with either fully dsDNA or partially duplexed DNA containing 3’ or 5’-overhangs in the presence of ATP. By incorporating a fluorophore on one DNA strand and a quencher in immediate proximity on the complementary strand, we were able to equate increased fluorescence to destabilization of the DNA duplex over time. Compared to the commercially available helicase, *Tte* UvrD, Hna exhibited no apparent DNA unwinding capabilities, regardless of the DNA substrate present (Fig. 3A)^48^. Additionally, using a colorimetric-based assay, we assessed the intrinsic and DNA-stimulated ability of Hna to hydrolyze ATP. These data revealed that Hna possesses minimal innate ATPase activity that is significantly enhanced by the inclusion of ssDNA (Supplementary Fig. 3A, 3B).

Given the pronounced relationship between Hna nuclease and ATPase activities, we wanted to investigate whether Hna uses distinct kinetic partitioning to self-regulate under physiological conditions. Kinetic partitioning is a strategy used by multifunctional enzymes to bias competing catalytic outcomes. This mechanism is commonly employed by DNA polymerases to maintain fidelity, but has also been characterized in bacterial defense systems such as the type I-D CRISPR-Cas system^49–51^. We speculated that the Hna nuclease and ATPase active sites may directly compete for resources or exhibit differential metal ion affinity to tune its active mode. To assess this, we performed additional ssDNA cleavage and ATPase assays while titrating the concentration of free Mg^2+^. These data revealed that Hna nuclease activity exhibits higher dependency on metal ion concentration than ATPase activity (Fig. 3B, C). Due to estimates of intracellular free Mg^2+^ levels ranging from ~0.3-1 mM^52^, we speculate that Hna likely exists in an ATP-bound state with direct inhibition of nuclease activity under native conditions.

To further characterize the direct competition between the nuclease and ATPase active sites, we performed mutagenesis on residues predicted to be involved in Mg^2+^ (P712A/D713A/E727A; *PDE Hna*) and ATP coordination (R36A/Q39A; *RQ Hna*) in the NUC and HEL1 domains, respectively (Fig. 3D, E). We speculated that disrupting the ability of each active site to chelate free metal ions would directly influence the dominant enzymatic mode. As predicted, reevaluation of ATPase activity revealed a significant increase in ATP hydrolysis by PDE Hna with total loss of activity by RQ Hna (Fig. 3F). Additionally, RQ Hna exhibited a 15-fold increase in ssDNA cleavage efficiency compared to wild-type Hna, while PDE Hna-mediated cleavage showed a marked decrease in product formation (Fig. 3G). Collectively, these data support a model where Hna employs kinetic partitioning between its ATPase and nuclease active sites. Disruption of Mg²⁺ coordination within the nuclease domain biases the enzyme toward ATP hydrolysis, whereas impairment of ATP coordination within the helicase domain shifts Hna toward enhanced DNA cleavage. The reciprocal enhancement of opposing activities in these mutants indicates that the two catalytic centers functionally compete, likely through shared metal ion utilization and/or allosteric coupling.

Lastly, we wanted to evaluate whether dysregulation of nuclease and ATPase function impact cell viability. We performed a series of cell toxicity assays following overexpression of each of our Hna mutants. These data revealed that mutants that exhibit enhanced nuclease activity (RQ Hna and Dimer mutant Hna) display increased cellular toxicity, whereas mutants with minimal nuclease activity (PDE Hna and dHna) show reduced toxicity relative to wild-type Hna (Fig. 3H, I). Together, these findings indicate that nuclease activity is the primary determinant of Hna-mediated cytotoxicity and support a model in which tight regulation of catalytic partitioning is required to prevent detrimental DNA degradation in vivo.

### Phage-encoded SSB activates Hna

To determine how Hna’s kinetic partitioning mechanism may be subverted during phage infection, we next sought to identify viral factors capable of disrupting the equilibrium of ATP-mediated nuclease inhibition. Previous work identified a predicted single-stranded DNA-binding protein (SSB) encoded by a T7-like podovirus, phage 5 A, that elicits an Hna-mediated immune response even in the absence of true phage infection. Additionally, escape phage carrying missense mutations of the 5 A SSB evaded detection by Hna^15^, implicating a potential interaction between Hna and the phage-encoded SSB leading to Hna-mediated abortive infection.

**Fig. 4:**
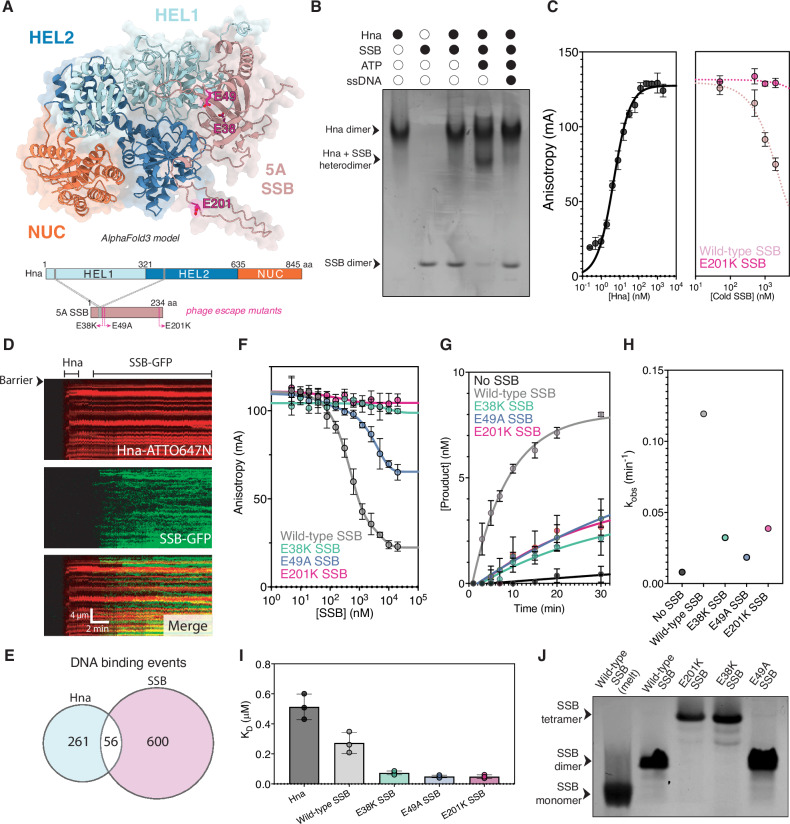
Phage-encoded 5 A SSB stimulates Hna nuclease activity. **A** AlphaFold3 structural prediction of Hna and phage-encoded, 5 A SSB heterodimer. Domain organization of each protein shown below with predicted protein-protein interface (gray) and characterized phage escape mutants (E38K, E49A, and E201K; dark pink). **B** Native gel shift assay to assess the basis of Hna and 5 A SSB complex formation. Hna and 5 A SSB allowed to dimerize independently then incubated at 37 °C for 30 minutes in the presence of a combination of ssDNA and/or ATP. Gel shown is representative of three independent experiments. **C** Fluorescence anisotropy of GFP–SSB (fixed concentration) was monitored during titration with increasing concentrations of unlabeled (cold) wild-type Hna in the presence of ATP (left). At saturating levels of Hna-SSB heterodimer formation, unlabeled wild-type or E201K SSB were added to assess competition for Hna binding (right). Data points represent mean ± SD from three biological replicates and were fit to a one site specific binding model. **D** Representative kymograph showing binding of ATTO647N-labeled Hna (red) and GFP-labeled 5 A SSB (green) on single-stranded DNA curtains in the presence of ATP. Hna was injected first, followed by the 5 A SSB to assess for colocalization events. **E** Counts of Hna (blue) and 5 A SSB (red) binding events on ssDNA curtains. Overlap in binding events (white) indicates instances of colocalization. **F** Fluorescence anisotropy starting with saturating levels of GFP-Hna homodimer followed by titration of cold, wild-type or 5 A SSB variants. Data points represent mean ± SD from three biological replicates and were fit to a one site specific binding model. **G**, Quantification of DNA cleavage by Hna in the presence of ATP and wild-type or 5 A SSB variants using capillary electrophoresis. Data shown are the mean ± standard deviation of three independent experiments for each condition, fit to a single or double-exponential equation. **H** Rates of Hna ssDNA cleavage incubated with various 5 A SSB variants, derived from fit of three independent experiments. **I** Binding affinity (K_D_) for ssDNA was determined for wild-type Hna and each 5 A SSB variant using fluorescence anisotropy and 6-FAM labeled ssDNA oligonucleotide. Data shown are individual binding affinities determined from three biological replicate binding curves, error bars represent the SD around the mean. **J** Native gel shift assay to evaluate the native stoichiometry of wild-type 5 A SSB and 5 A SSB escape mutants. A portion of the wild-type SSB reaction was melted at 95 °C for 10 minutes to visualize the monomeric state. Gel shown is representative of three independent experiments. Source data are provided as a Source Data file.

Structural predictions revealed that the 5 A SSB bears similarity to another SSB, gp2.5, encoded by bacteriophage T7^53,54^. Based on this comparison, we suspected that the 5 A SSB likely forms a stable dimer without DNA; however, highest likelihood predictions provided by AF3 suggest that Hna and the 5 A SSB form a heterodimer composed of one copy of each protein (Fig. 4A)^41^. Formation of an Hna and 5 A SSB complex would, therefore, necessitate destabilization of the Hna and 5 A SSB dimers in favor of heterodimer assembly. To test this, we first performed a native gel shift assay using Hna, 5 A SSB, ssDNA, and ATP after pre-assembling each component into its dimeric form. We observed that a unique species forms in the presence of Hna, 5 A SSB, and ATP, but not ssDNA, that is consistent in size with an Hna and 5 A SSB heterodimer (Fig. 4B).

To more extensively assess Hna-SSB heterodimer formation, we performed additional fluorescence anisotropy experiments using GFP-tagged 5 A SSB in the presence of ATP. Holding the concentration of GFP-SSB constant and titrating unlabeled Hna, we observed a concentration-dependent increase in anisotropy (Fig. 4C). At saturating levels of heterodimer formation, we then introduced unlabeled 5 A SSB as a competitor to promote dissolution of the labeled complex. As expected, we observed decreased binding as a direct function of cold competitor concentration, indicating that Hna readily interacts with the phage-encoded SSB (Fig. 4C).

Surprisingly, Hna and the 5 A SSB appeared to interact independent of ssDNA (Fig. 4B). To directly visualize Hna DNA interrogation we used single-molecule ssDNA curtains with Hna fluorescently labeled with ATTO647N, as previously described^55,56^, and GFP-SSB in the absence and presence of ATP. We observed very few, randomly distributed Hna binding events along the length of the ssDNA curtains. Upon introduction of ATP, Hna ssDNA binding was significantly enriched, establishing that Hna functions predominantly as an ATP-dependent single-stranded DNA binding protein (Supplementary Fig. 4A). Interestingly, in the absence of buffer flow, Hna rapidly diffuses along the DNA independent of ATP (Supplementary Fig. 4B). Lack of ATP-dependent translocase activity suggests that ATP binding assists in the localization of Hna to ssDNA targets but does not contribute to the innate ability of Hna to diffuse rapidly along lengths of ssDNA. Although we observed extensive Hna and 5 A SSB binding along the lengths of the DNA molecules, we did not record consistent or significant colocalization of the two proteins. This observation supports a model in which Hna and the 5 A SSB form a complex prior to, or independent of, DNA binding (Fig. 4D, E).

Given that Hna likely exists as an autoinhibited dimer under physiological conditions, we questioned whether introduction of the 5 A SSB was sufficient to destabilize this dimeric state. To assess this we performed additional fluorescence anisotropy experiments using saturating levels of GFP-Hna homodimer complex. We then titrated unlabeled 5 A SSB and observed a significant decrease in anisotropy in the presence of ATP, suggesting the phage-encoded SSB promotes dissolution of the autoinhibited Hna dimer (Fig. 4F). We then performed additional ssDNA cleavage experiments in the presence of 5 A SSB and observed a substantial increase in nuclease activity despite the inclusion of ATP (Fig. 4G, Supplementary Fig. 4C). Together, these findings demonstrate that the 5 A SSB is sufficient to destabilize the autoinhibited Hna homodimer and to relieve ATP-dependent repression of nuclease activity. The concomitant increase in ssDNA cleavage suggests that SSB-mediated dimer destabilization shifts Hna out of its ATPase-dominant state and toward nuclease activation.

We next wanted to assess whether the 5 A SSB escape mutants could elicit similar effects on dysregulation of Hna nuclease activity. When evaluating the effect of each 5 A SSB mutant on Hna homodimer dissolution and Hna ssDNA cleavage efficiency, we observed that the mutant SSB’s exhibited significantly reduced to no effect on Hna dimer disruption and only marginally increased DNA degradation compared to the no SSB control (Fig. 4F–H). Collectively, these findings indicate that the escape mutations impair the ability of the 5 A SSB to disrupt the autoinhibited Hna homodimer, thereby preventing heterodimer formation, nuclease activation, and evading Hna surveillance.

Closer inspection of the AF3 model of the Hna and 5 A SSB complex suggests that Hna and 5 A SSB monomers compete for the same domain along the Hna dimerization interface; however, upon mapping each of the previously studied 5 A SSB escape mutants, we noted that none of the mutated residues are within the dimerization interface (Fig. 4A). This provides a structural rationale for how the wild-type phage-encoded SSB may promote disassembly of the Hna homodimers but does not provide an intuitive explanation for how the escape mutants evade Hna detection. Due to each of the missense mutations conferring loss of a negatively charged residue, we speculated that the 5 A SSB mutants may exhibit enhanced DNA binding that promotes localization to the phage DNA without being sequestered by Hna. We used fluorescence anisotropy to measure the binding affinity (K_d_) of Hna, wild-type 5 A SSB, and three of the reported 5 A SSB mutants (E38K, E49A, and E201K) for a single-stranded DNA substrate in the presence of ATP. As predicted, each of the 5 A SSB mutants exhibited improved DNA binding compared to wild-type 5 A SSB, with Hna possessing the weakest affinity for ssDNA even in the presence of ATP (Fig. 4I, Supplementary Fig. 4E). Additionally, native gel shift experiments revealed that two of the three 5 A SSB mutants, E38K and E201K, adopt a unique, tetrameric stoichiometry in the absence of DNA as opposed to the expected dimeric state (Fig. 4J). The persistence of the E49A SSB mutant as a dimer may explain its ability to still moderately disrupt the Hna homodimer (Fig. 4F). Collectively, these data demonstrate that the 5 A SSB mutants utilize diverse strategies to evade Hna detection through adopting higher order conformations that likely perturb formation of the Hna-SSB complex and increased affinity for ssDNA, further obstructing Hna-mediated anti-phage action.

## Discussion

Hna is an abortive infection, bacterial immune system that provides protection from phage through a previously undetermined mechanism. Here, we show that Hna functions as a 3’—5’ ssDNA exonuclease that is negatively regulated through the formation of an auto-inhibited homodimer under physiologically relevant concentrations of ATP. Hna dimers can be destabilized through the incorporation of phage-derived SSBs, stimulating dysregulated nuclease activity that likely contributes to host cell death or dormancy. Overall, these data support that Hna is a broadly distributed anti-phage system that can surveil for phage-encoded SSB, resulting in constitutive exonuclease activation to confer resistance against phage infection.

SF2 helicases typically couple ATP binding and hydrolysis to directional translocation or duplex unwinding^57^. In contrast, Hna uses ATP to restrain, rather than stimulate, DNA cleavage. Both ATP and the non-hydrolyzable analog ADP·BeF₃ suppress nuclease activity, indicating that ATP binding, rather than hydrolysis, is sufficient to promote stabilization of the inhibited state. Cryo-EM analysis revealed that Hna assembles into a homodimer with pseudo-C2 symmetry via the HEL1 domain, accompanied by pronounced flexibility within the C-terminal NUC domain. This rearrangement likely disrupts productive alignment of catalytic residues and provides a structural basis for ATP-dependent repression during non-infection states. Although ATP hydrolysis stimulated by ssDNA binding may promote dimer dissolution, due to low availability of ssDNA under non-stressed conditions^58,59^, Hna likely remains unbound and trapped in a nuclease-incompetent state while acting as a surveillance system for phage-encoded factors, such as SSBs. While we acknowledge that Hna possesses basal levels of intrinsic ATPase activity, this process is exceptionally slow and likely favors dimer reformation in the case of spontaneous dissolution^57,60^.

Our kinetic analyses demonstrate that Hna catalytic outputs are governed by kinetic partitioning between its ATPase and nuclease domains. Nuclease activity exhibits stronger dependence on free Mg²⁺ than ATPase activity, suggesting that intracellular Mg²⁺ levels bias Hna toward an ATP-bound, cleavage-suppressed state. Reciprocal active-site mutations further reinforce this model and demonstrate that disruption of nuclease metal coordination enhances ATP hydrolysis, whereas impairment of ATP coordination markedly stimulates DNA cleavage. The inverse relationship between these activities indicates functional competition, potentially through shared metal utilization or allosteric coupling. While PD-(D/E)XK nucleases often employ a two metal ion mechanism of DNA cleavage, several instances of three metal ion coordination have been reported within this family and provide additional structural support necessary for catalysis^61–63^. We speculate that the increased sensitivity of Hna nuclease activity on metal ion concentrations may be attributed to additional ion coordination requirements. Domain rearrangements upon ATP binding and subsequent dimer formation may also disrupt or weaken metal ion binding within the NUC active site, providing an explanation for the lack of exonuclease activity and increased flexibility of the NUC domain in our Hna dimer structure.

**Fig. 5:**
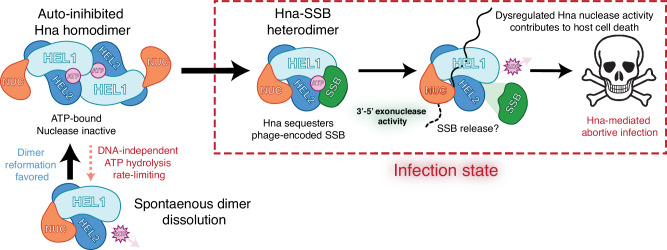
Proposed mechanism of Hna-mediated anti-phage action. Hna forms an auto-inhibited homodimer in the presence of ATP. Intrinsic ATPase activity contributing to dimer dissolution is likely slow and rate-limiting due to the low availability of single-stranded DNA during non-infection states. Upon phage infection, phage-encoded protein factors, such as single-stranded DNA binding proteins, can complex with Hna, releasing Hna from auto-inhibition, and stimulating Hna nuclease activity. Dysregulated DNA degradation by Hna during infection likely results in degradation of host plasmid or loss in host genome integrity, leading to abortive infection.

Our work also provides direct biochemical evidence of Hna nuclease activation mediated by a phage-encoded SSB. Structural modeling predicts that Hna and 5 A SSB form a heterodimer through competition at the Hna dimerization interface. Biochemical analyses confirm that wild-type 5 A SSB destabilizes the autoinhibited Hna homodimer and relieves nuclease repression, thereby shifting catalytic partitioning toward DNA cleavage. Single-molecule imaging indicates that Hna and 5 A SSB do not require DNA to interact, supporting a model in which heterodimer formation precedes or occurs independently of DNA engagement. Functionally, this interaction stimulates nuclease activation even in the presence of ATP, possibly resulting in loss of host genome integrity or unregulated degradation of host plasmid, leading to abortive infection (Fig. 5). We therefore propose that 5 A SSB acts as a molecular trigger that converts Hna from a surveillance mode to an abortive infection effector. Intriguingly, escape mutations within 5 A SSB fail to disrupt the Hna homodimer and do not robustly stimulate nuclease activity. Instead, escape mutants exhibit increased ssDNA binding and adopt higher-order oligomeric states in the absence of DNA. These changes likely favor sequestration of phage DNA while simultaneously reducing productive engagement with Hna.

Hna functions as a single-effector ssDNA exonuclease with highly-conserved SF2 helicase domains involved in ATP hydrolysis and dimerization. We describe modes of Hna auto-inhibition and activation by a phage-encoded SSB. This mechanism illustrates how ATP-dependent oligomerization and metal-sensitive catalytic partitioning can be integrated into a tunable immune switch. More broadly, our findings expand the functional repertoire of SF2 helicase-associated nucleases and highlight kinetic partitioning as a versatile strategy for balancing immune activation with host viability. Future work is needed to resolve discrete modes of DNA-binding and catalysis by Hna. We anticipate that continued investigation of Hna orthologs will reveal diversity in function and recognition of phage-encoded factors across members of the Hna family of bacterial immune systems.

## Methods

### Plasmid construction

*S. meliloti* Hna and phage-encoded 5 A SSB protein sequences as previously reported^15^ were reverse translated and codon optimized for expression in *E. coli*. The full-length Hna gene was commercially synthesized by Integrated DNA Technology (IDT) as a gBlock and assembled into a pET28 expression vector with a 6X N-terminal His tag, MBP tag, and TEV protease site using Gibson Assembly. Point mutations were introduced using site directed mutagenesis to generate each of the Hna variants. Each of the 5 A SSB variants was commercially cloned into a pET28 expression vector with a 6X N-terminal His tag by Twist Biosciences.

The constructs described above were further modified for use in single-molecule fluorescence microscopy and fluorescence anisotropy experiments. We ordered the coding sequence for a 3X-FLAG tag as a gBlock from IDT, then inserted it directly upstream of the Hna gene using Gibson Assembly. Additionally, we ordered a coding sequence for GFP as a gBlock from IDT and inserted it directly downstream of the Hna or 5 A SSB gene using Gibson Assembly. These constructs were used to produce the Hna and SSB protein used for all single-molecule fluorescence microscopy experiments.

### Protein expression and purification

All Hna and 5 A SSB proteins were recombinantly expressed and purified from *E. coli*. The expression plasmids described above were transformed into BL21 (DE3) or C41 (DE3) cells, plated on LB medium plates, and grown overnight at 37 °C. Single colonies were used to inoculate 30 mL of LB media and cultured overnight at 37 °C. For each liter of LB media, 10 mL of overnight starter culture was used to inoculate the expression cultures. Expression cultures were allowed to grow (37 °C, 220 rpm) until reaching an optical density at 600 nm (OD_600_) of 0.6-0.7, then induced with 0.5 mM isopropyl-β-d-1-thiogalactopyranoside (IPTG) for 18 hours at 16°C (180 rpm).

Following induction, cells were pelleted by centrifugation and lysed via sonication in buffer containing 20 mM Tris-HCl (pH 7.5), 10% glycerol, 500 mM NaCl, 0.5 mM tris(2-carboxyethyl)phosphine (TCEP), 10 mM MgCl_2_, 1X DNase, and Pearce protease inhibitor tablets (Thermo Scientific). The cell lysate was clarified via ultracentrifugation and loaded onto a HisTrap HP column equilibrated in buffer containing 20 mM Tris-HCl (pH 7.5), 10% glycerol, 500 mM NaCl, and 0.5 mM TCEP, and eluted in buffer containing 20 mM Tris-HCl (pH 7.5), 10% glycerol, 500 mM NaCl, 0.5 mM TCEP, and 250 mM imidazole (pH 8). Fractions containing the target protein were added to Spectra/Por dialysis tubing (12-14 kD MWCO) and incubated in buffer containing 20 mM HEPES (pH 7.5), 10% glycerol, 150 mM KCl, and 0.5 mM TCEP at 4°C overnight. For Hna proteins, TEV protease was added for removal of affinity and MBP tags during dialysis. Dialyzed protein was concentrated using a 10 kDa or 50 kDa MWCO centrifugal filter (Sigma-Aldrich) and purified by size-exclusion chromatography (Superose 6 10/300; GE Healthcare) in buffer containing 20 mM HEPES (pH 7.5), 10% glycerol, 150 mM KCl, and 0.5 mM TCEP. Purified proteins were concentrated, aliquoted, flash-frozen using liquid nitrogen, and stored at -80°C.

### DNA cleavage assay

Hna cleavage assays were conducted using a buffer containing 20 mM Tris-HCl (pH 7.5), 100 mM KCl, 10 mM MgCl_2_, and 5% glycerol with 100 nM protein and 10 nM DNA. For select experiments, ATP or ADP·BeF_3_ were added at 1 mM unless otherwise specified. All DNA substrates tested are listed in Supplementary Table 1. Reactions were incubated at 37 °C for the indicated time and terminated with the addition of 50 mM EDTA. For gel-based assays, Proteinase K and SDS were individually added to each reaction, mixed with TBE-Urea Sample Buffer (Novex), loaded onto a 15% TBE-Urea PAGE gel, and DNA products were allowed to separate for 2 hours at 90 V. Gels were imaged using a fluorescence scanner. For analysis via capillary electrophoresis, reactions were combined with Hi-Di formamide (Applied Biosystems) and processed using a 3730 series DNA Analyzer (Applied Biosystems). Peaks corresponding to unique DNA species and sizes were quantified using the GeneMapper software.

### Cryo-EM sample preparation and data collection

To determine a structure of an Hna monomer, 15 µM Hna was incubated with 10 mM CaCl_2_ and 10 µM 3’-PS single-stranded DNA for 5 minutes at 37 °C. 2.5 µL of sample was applied to glow discharged holey carbon grids (Quantifoil 1.2/1.3), blotted for 8 seconds with a blot force of 0, at 4°C and 100% humidity, then rapidly plunged into liquid ethane using an FEI Vitrobot Mark IV (Thermo Fisher). For the dimeric Hna structure, 15 µM Hna was incubated with 10 mM MgCl_2_, 10 µM 3’-PS ssDNA, and 1 mM ADP·BeF_3_ for 20 minutes at 37 °C. 2.5 µL of sample was applied to glow discharged holey carbon grids (Quantifoil 1.2/1.3), blotted for 7 seconds with a blot force of 1, at 4°C and 100% humidity, then rapidly plunged into liquid ethane using an FEI Vitrobot Mark IV (Thermo Fisher). For both structures, data was collected using a FEI Glacios cryo-EM microscope (200 kV) equipped with a Falcon 4 direct electron detector (Gatan). Movies were recorded in SerialEM with a pixel size of 0.94 Å and a total exposure time of 15 seconds for an accumulated dose of 49 e^-^/Å^2^.

### Cryo-EM data processing and model building

All cryo-EM data processing was performed using CryoSPARC. Particles were initially picked by applying a minimum and maximum particle diameter of 70 and 100 Å, respectively for the monomeric dataset, and 70 and 150 Å, respectively for the dimeric dataset. Particle picks were manually inspected to eliminate particle outliers.

For the Hna monomer dataset, particles were extracted with a box size of 300 pixels with a fourier crop to 128 pixels and then classified into 50 2D classes. 2D classes were manually selected then processed through one round of ab-initio volume reconstruction and heterogeneous refinement. The representative 3D class was further refined using non-uniform refinement. The resulting volume was used to generate 50, 2D templates that were used to guide particle picking via template picker. The refined set of particles was manually inspected and extracted with a box size of 284 pixels with a fourier crop to 128 pixels and then classified into 50 2D classes. 2D classes were manually selected then processed through two rounds of ab-initio volume reconstruction and heterogenous refinement with 5 classes for each. The representative 3D class from the final round of heterogenous refinement was further processed using non-uniform refinement, particles were unbinned, then underwent a final round of non-uniform refinement to determine a 3.9 Å structure of an Hna monomer.

For the Hna dimer dataset, particles were extracted with a box size of 320 pixels with a fourier crop of 128 pixels and then classified into 50 2D classes. 2D classes were manually selected then processed through one round of ab-initio volume reconstruction and heterogeneous refinement. The representative 3D class was further refined using non-uniform refinement and then evaluated for particle heterogeneity using 3D classification. Volumes representing the same consensus structure were combined and refined using non-uniform refinement. Particles were unbinned and over-represented views were removed using the rebalance orientations job. The final set of particles was input into a reconstruct only job to determine a 4.4 Å structure of an Hna dimer.

Both Hna structures were initially rigid body fit using models generated from AlphaFold3 predictions within ChimeraX. For the Hna monomer, domain conformations were individually rigid body fit, then further refined through interactive rounds of modeling in Isolde (version 1.10) and Coot (version 0.9.8.95). The model was subsequently processed using real space refinement in Phenix (version 1.21) to generate final validation scores. Detailed structural analysis pipeline and validation statistics for both structures provided in Supplementary Fig. 5 and Supplementary Table 2, respectively.

### DNA unwinding assay

Double-stranded DNA duplexes were prepared by combining either a 3’ or 5’-fluorescently labeled ssDNA oligonucleotide with a complementary ssDNA containing either a 3’ or 5’-Black Hole Quencher (BHQ-1) in a 1:2 molar ratio. Each mixture was heated to 95°C for 2 minutes and then slowly cooled at room temperature over the span of 4 hours. The DNA unwinding assay was performed by combining 100 nM of Hna with 10 nM of fully duplexed DNA or partially duplexed DNA containing either a 3’ or 5’ ssDNA overhang in a buffer containing 20 mM Tris-HCl (pH 7.5), 100 mM KCl, 10 mM MgCl_2_, 5% glycerol, and 1 mM ATP. Reactions were monitored in real time at 37 °C for up to 90 minutes in the presence of an unlabeled strand identical to the FAM-labeled strand at 10X molar surplus to sequester the complementary strand upon DNA unwinding. Due to fluorescence quenching while duplexed, we monitored for increases in fluorescence over time using a CLARIOstar Plus plate reader (BMG Labtech).

### ATPase assay

Hna ATPase activity was assessed using a Malachite green phosphate assay kit (Sigma-Aldrich). Phosphate standards were prepared as detailed by the kit. Reactions were conducted using 100 nM Hna and a reaction buffer containing 20 mM Tris-HCl (pH 7.5), 100 mM KCl, 10 mM MgCl_2_, 5% glycerol, and 20 µM ATP, ADP·BeF_3_, AMP-PNP, ADP, CTP, or GTP with or without the inclusion of 10 nM unlabeled ssDNA. Each reaction was performed in triplicate and incubated at 37 °C for 20 minutes. Reagent provided by the kit was added to each reaction, incubated at room temperature for 30 minutes, and then absorbance values at 620 nm were recorded for each reaction using a CLARIOstar Plus plate reader (BMG Labtech). We determined the concentration of free phosphate produced in each reaction by converting the raw fluorescence values to concentration using the standard curve generated.

### Electrophoretic mobility shift assay

To evaluate the basis of Hna dimerization, 1 µM Hna was pre-incubated for up to 2 hours at 37 °C in a buffer containing 20 mM Tris-HCl (pH 7.5), 100 mM KCl, 10 mM MgCl_2_, and 5% glycerol with 10 nM ssDNA and/or 1 mM ATP. Reactions were loaded onto a non-denaturing 4-20% Tris-glycine protein gel (Invitrogen) and run at 100 V for 90 minutes at 4°C. For assessing Hna and SSB interaction and SSB stoichiometry, each protein was allowed to independently oligomerize for 1 hour prior to initiating the reaction. Hna and SSB were combined in equimolar concentration using the buffer composition described above in the presence or absence of 10 nM ssDNA and/or 1 mM ATP. Reactions were loaded onto a non-denaturing 4-20% Tris-glycine protein gel and run at 100 V for 45-90 minutes at 4°C.

### Fluorescence anisotropy

For Hna and SSB oligomerization assays, GFP-Hna or SSB were included at a concentration of 10 nM in a buffer containing 20 mM Tris-HCl (pH 7.5), 100 mM KCl, 10 mM MgCl_2_, 5% glycerol, and 0.05% Tween-20. We performed a series of 2-fold dilutions to titrate the concentration of cold Hna, SSB, or competitor based on the given experiment. For disassembly experiments, saturating levels of labeled complex were pre-formed prior to introduction of cold competitors. Each reaction was incubated for 30 minutes at 37 °C in the presence or absence of ATP, then anisotropy values were recorded using a CLARIOstar Plus plate reader (BMG Labtech) equipped with a fluorescence polarization optical module. Data were fit using a one-step binding model to define the K_D_ for each unique protein tested.

For DNA binding assays, Hna or 5 A SSB were included at the highest concentration possible in a buffer containing 20 mM Tris-HCl (pH 7.5), 100 mM KCl, 10 mM MgCl_2_, 5% glycerol, and 0.05% Tween-20. We performed a series of 2-fold dilutions of each protein in triplicate, then combined each dilution with 10 nM of FAM-labeled single-stranded DNA with 3’-phosphorothioate modifications to observe binding without degradation. Each reaction was incubated for 30 minutes at 37 °C in the presence or absence of ATP, then anisotropy values were recorded using a CLARIOstar Plus plate reader (BMG Labtech) equipped with a fluorescence polarization optical module. Data were fit using a one-step binding model to define the K_D_ for each unique protein tested.

### Cellular toxicity assay

BL21(DE3)-competent cells containing recombinant plasmids for various Hna variants were grown up to an OD_600_ of approximately 0.6 at 37 °C. The culture was then split into two equal volumes: (1) as an uninduced negative control and (2) the other induced with a final concentration of 0.5 mM IPTG. Both cultures with or without IPTG were incubated for 18 hours at 16 °C. A series of eight, tenfold dilutions were made for both the cultures with and without IPTG and were plated on dry LB agar plates containing antibiotics for Hna recombinant plasmid. The LB agar plates were incubated at 37 °C overnight. Colonies were quantified from the dilution series to measure colony growth repression due to Hna toxicity arresting cell growth.

### Single-molecule fluorescence microscopy

Single-molecule fluorescent images were collected using a customized prism TIRF microscopy-based inverted Nikon Ti-E microscope system equipped with a motorized stage (Prio ProScan II H117). The sample was illuminated with a 488-nm laser (Coherent Sapphire) and a 637-nm laser (Coherent OBIS) split by a 638-nm dichroic beam splitter (Chroma). Two color imaging was recorded using dual electron-multiplying charge-coupled device (EMCCD) cameras (Andor iXon DU897). Subsequent files were exported as uncompressed TIFF stacks and further analyzed in FIJI. Flowcells used for single-molecule DNA experiments were prepared as previously described^55,56^. All the single-molecule experiments were conducted in the imaging buffer (40 mM Tris-HCl pH 8.0, 50 mM NaCl, 2 mM MgCl2, 1 mM DTT and 0.2 mg mL/1 BSA) with or without 1 mM ATP at 37 °C.

For the fluorescent labeling of Hna, protein was conjugated with monoclonal anti-Flag antibody (Sigma Aldrich, F1804) and anti-Mouse-IgG-ATTO647N antibody produced in goat (Sigma Aldrich, 50185) on ice for 10 minutes. The mixture was then diluted to a total volume of 100 μL imaging buffer with 200 nM final protein injection concentration. Immediately after the conjugation and dilution, the fluorescently labeled protein, Hna-ATTO647, was injected into the flowcell at a 0.12 mL min-1 flow rate. During image acquisition, the exposure time was set to 60 ms, with an interval of 1–2 seconds between frames. To observe Hna diffusion events, flow was temporarily turned off during imaging.

To image the binding of Hna on ssDNA and its colocalization with 5 A SSB-GFP, 100 μL volume of Hna-ATTO647 protein (final concentration 200 nM) in ATP imaging buffer was injected into the flowcell, followed by injection of SSB-GFP (final concentration 1 nM) in ATP imaging buffer using 10 mL syringe for at least 10 minutes.The buffer flow was set to 0.2 mL min-1 flow rate. During image acquisition, the exposure time was set to 60 ms, with an interval of 2 seconds between frames.

### Reporting summary

Further information on research design is available in the Nature Portfolio Reporting Summary linked to this article.

## Supplementary information

Supplementary Information

Reporting Summary

Transparent Peer Review file

## Source data

Source Data

## Data Availability

The structures and associated atomic coordinates have been deposited into the Electron Microscopy Data Bank (EMDB) and Protein Data Bank (PDB) with accession codes: Hna Monomer (EMD-75470 [https://www.ebi.ac.uk/emdb/EMD-75470] and PDB 10UJ) and Hna Dimer (EMD-73047 [https://www.ebi.ac.uk/emdb/EMD-73047] and PDB 9YKJ). Source data are provided with this paper.

## References

[1] Ranveer, S. A. et al. Positive and negative aspects of bacteriophages and their immense role in the food chain. *npj Sci. Food* **8**, 1 (2024). 38172179 10.1038/s41538-023-00245-8 PMC10764738

[2] Lang, A. S., Buchan, A. & Burrus, V. Interactions and evolutionary relationships among bacterial mobile genetic elements. *Nat. Rev. Microbiol* **23**, 423–438 (2025). 40069292 10.1038/s41579-025-01157-y

[3] Doron, S. et al. Systematic discovery of antiphage defense systems in the microbial pangenome. *Science* **359**, eaar4120 (2018). 29371424 10.1126/science.aar4120 PMC6387622

[4] Millman, A. et al. An expanded arsenal of immune systems that protect bacteria from phages. *Cell Host Microbe* **30**, 1556–1569.e5 (2022). 36302390 10.1016/j.chom.2022.09.017

[5] Bernheim, A. & Sorek, R. The pan-immune system of bacteria: antiviral defence as a community resource. *Nat. Rev. Microbiol* **18**, 113–119 (2020). 31695182 10.1038/s41579-019-0278-2

[6] Vassallo, C. N., Doering, C. R., Littlehale, M. L., Teodoro, G. I. C. & Laub, M. T. A functional selection reveals previously undetected anti-phage defence systems in the E. coli pangenome. *Nat. Microbiol* **7**, 1568–1579 (2022). 36123438 10.1038/s41564-022-01219-4 PMC9519451

[7] Jiang, F. & Doudna, J. A. CRISPR–Cas9 Structures and Mechanisms. *Annu. Rev. Biophys.* **46**, 505–529 (2017). 28375731 10.1146/annurev-biophys-062215-010822

[8] Nussenzweig, P. M. & Marraffini, L. A. Molecular Mechanisms of CRISPR-Cas Immunity in Bacteria. *Annu. Rev. Genet.* **54**, 93–120 (2020). 32857635 10.1146/annurev-genet-022120-112523

[9] Lopatina, A., Tal, N. & Sorek, R. Abortive Infection: Bacterial Suicide as an Antiviral Immune Strategy. *Annu. Rev. Virol.* **7**, 371–384 (2020). 1. 32559405 10.1146/annurev-virology-011620-040628

[10] Aframian, N. & Eldar, A. Abortive infection antiphage defense systems: separating mechanism and phenotype. *Trends Microbiol.* **31**, 1003–1012 (2023). 37268559 10.1016/j.tim.2023.05.002

[11] Kelly, A., Arrowsmith, T. J., Went, S. C. & Blower, T. R. Toxin–antitoxin systems as mediators of phage defence and the implications for abortive infection. *Curr. Opin. Microbiol.* **73**, 102293 (2023). 36958122 10.1016/j.mib.2023.102293

[12] Jaskólska, M., Adams, D. W. & Blokesch, M. Two defence systems eliminate plasmids from seventh pandemic Vibrio cholerae. *Nature* **604**, 323–329 (2022). 35388218 10.1038/s41586-022-04546-y PMC7613841

[13] Deep, A., Liang, Q., Enustun, E., Pogliano, J. & Corbett, K. D. Architecture and activation mechanism of the bacterial PARIS defence system. *Nature* **634**, 432–439 (2024). 39112702 10.1038/s41586-024-07772-8 PMC11479591

[14] Cui, Y. et al. Bacterial Hachiman complex executes DNA cleavage for antiphage defense. *Nat. Commun.* **16**, 2604 (2025). 40097437 10.1038/s41467-025-57851-1 PMC11914072

[15] Sather, L. M. et al. A broadly distributed predicted helicase/nuclease confers phage resistance via abortive infection. *Cell Host Microbe* **31**, 343–355.e5 (2023). 36893733 10.1016/j.chom.2023.01.010

[16] Song, X. et al. Catalytically inactive long prokaryotic Argonaute systems employ distinct effectors to confer immunity via abortive infection. *Nat. Commun.* **14**, 6970 (2023). 37914725 10.1038/s41467-023-42793-3 PMC10620215

[17] Tesson, F. et al. Systematic and quantitative view of the antiviral arsenal of prokaryotes. *Nat. Commun.* **13**, 2561 (2022). 35538097 10.1038/s41467-022-30269-9 PMC9090908

[18] Néron, B. et al. MacSyFinder v2: Improved modelling and search engine to identify molecular systems in genomes. *Peer Community Journal* **3**, (2023).

[19] Tesson, F. et al. A Comprehensive Resource for Exploring Antiphage Defense: DefenseFinder Webservice,Wiki and Databases. *Peer Community Journal* **4**, (2024)

[20] Tuck, O. T. et al. Genome integrity sensing by the broad-spectrum Hachiman antiphage defense complex. *Cell* **187**, 6914–6928.e20 (2024). 39395413 10.1016/j.cell.2024.09.020 PMC12278908

[21] Huo, Y. et al. Structural and biochemical insights into the mechanism of the Gabija bacterial immunity system. *Nat. Commun.* **15**, 836 (2024). 38282040 10.1038/s41467-024-45173-7 PMC10822852

[22] Wu, Y. et al. Bacterial defense systems exhibit synergistic anti-phage activity. *Cell Host Microbe* **32**, 557–572.e6 (2024). 38402614 10.1016/j.chom.2024.01.015 PMC11009048

[23] Cheng, R. et al. Prokaryotic Gabija complex senses and executes nucleotide depletion and DNA cleavage for antiviral defense. *Cell Host Microbe* **31**, 1331–1344.e5 (2023). 37480847 10.1016/j.chom.2023.06.014

[24] Bari, S. M. N. et al. A unique mode of nucleic acid immunity performed by a multifunctional bacterial enzyme. *Cell Host Microbe* **30**, 570–582.e7 (2022). 35421352 10.1016/j.chom.2022.03.001

[25] Jackson, R. N., Lavin, M., Carter, J. & Wiedenheft, B. Fitting CRISPR-associated Cas3 into the Helicase Family Tree. *Curr. Opin. Struct. Biol.* **24**, 106–114 (2014). 24480304 10.1016/j.sbi.2014.01.001 PMC3984625

[26] Depardieu, F. et al. A Eukaryotic-like Serine/Threonine Kinase Protects Staphylococci against Phages. *Cell Host Microbe* **20**, 471–481 (2016). 27667697 10.1016/j.chom.2016.08.010

[27] Garb, J. et al. Multiple phage resistance systems inhibit infection via SIR2-dependent NAD+ depletion. *Nat. Microbiol* **7**, 1849–1856 (2022). 36192536 10.1038/s41564-022-01207-8

[28] Gao, L. A. et al. Prokaryotic innate immunity through pattern recognition of conserved viral proteins. *Science* **377**, eabm4096 (2022). 35951700 10.1126/science.abm4096 PMC10028730

[29] Zhang, T. et al. Direct activation of a bacterial innate immune system by a viral capsid protein. *Nature* **612**, 132–140 (2022). 36385533 10.1038/s41586-022-05444-z PMC9712102

[30] Stokar-Avihail, A. et al. Discovery of phage determinants that confer sensitivity to bacterial immune systems. *Cell* **186**, 1863–1876.e16 (2023). 37030292 10.1016/j.cell.2023.02.029

[31] Sasaki, T. et al. Phage single-stranded DNA-binding protein or host DNA damage triggers the activation of the AbpAB phage defense system. *mSphere* **8**, e00372-23 (2023). 37882551 10.1128/msphere.00372-23 PMC10732053

[32] Fairman-Williams, M. E., Guenther, U.-P. & Jankowsky, E. SF1 and SF2 helicases: family matters. *Curr. Opin. Struct. Biol.* **20**, 313–324 (2010). 20456941 10.1016/j.sbi.2010.03.011 PMC2916977

[33] Steczkiewicz, K., Muszewska, A., Knizewski, L., Rychlewski, L. & Ginalski, K. Sequence, structure and functional diversity of PD-(D/E)XK phosphodiesterase superfamily. *Nucleic Acids Res*. **40**, 7016–7045 (2012). 22638584 10.1093/nar/gks382 PMC3424549

[34] Cheng, K. Structure, function and evolution of the bacterial DinG-like proteins. *Comput Struct. Biotechnol. J.* **27**, 1124–1139 (2025). 40206346 10.1016/j.csbj.2025.03.023 PMC11981726

[35] Gao, T. et al. Structural and functional investigation of DinG containing a 3′–5′ exonuclease domain. *mBio* **16**, e00884-25 (2025). 40586552 10.1128/mbio.00884-25 PMC12345164

[36] Fang, T., Wang, X. & Huangfu, N. Superfamily II helicases: the potential therapeutic target for cardiovascular diseases. *Front. Cardiovasc. Med*. **10**, (2023). 10.3389/fcvm.2023.1309491 PMC10752008 38152606

[37] Liu, H. et al. Structure of the DNA repair helicase XPD. *Cell* **133**, 801–812 (2008). 18510925 10.1016/j.cell.2008.04.029 PMC3326533

[38] White, M. F. Structure, function and evolution of the XPD family of iron–sulfur-containing 5′→3′ DNA helicases. *Biochem Soc. Trans.* **37**, 547–551 (2009). 19442249 10.1042/BST0370547

[39] Enemark, E. J. & Joshua-Tor, L. On Helicases and other motor proteins. *Curr. Opin. Struct. Biol.* **18**, 243–257 (2008). 18329872 10.1016/j.sbi.2008.01.007 PMC2396192

[40] Knizewski, L., Kinch, L. N., Grishin, N. V., Rychlewski, L. & Ginalski, K. Realm of PD-(D/E)XK nuclease superfamily revisited: detection of novel families with modified transitive meta profile searches. *BMC Struct. Biol.* **7**, 40 (2007). 17584917 10.1186/1472-6807-7-40 PMC1913061

[41] Abramson, J. et al. Accurate structure prediction of biomolecular interactions with AlphaFold 3. *Nature* **630**, 493–500 (2024). 38718835 10.1038/s41586-024-07487-w PMC11168924

[42] Tanner, N. K., Cordin, O., Banroques, J., Doère, M. & Linder, P. The Q Motif: A Newly Identified Motif in DEAD Box Helicases May Regulate ATP Binding and Hydrolysis. *Mol. Cell* **11**, 127–138 (2003). 12535527 10.1016/s1097-2765(03)00006-6

[43] Chabre, M. Aluminofluoride and beryllofluoride complexes: new phosphate analogs in enzymology. *Trends Biochemical Sci.* **15**, 6–10 (1990). 10.1016/0968-0004(90)90117-t 2180149

[44] Chen, M. C. & Ferré-D’Amaré, A. R. Structural Basis of DEAH/RHA Helicase Activity. *Crystals* **7**, 253 (2017).

[45] Yaginuma, H. et al. Diversity in ATP concentrations in a single bacterial cell population revealed by quantitative single-cell imaging. *Scientific Reports* **4**, (2014). 10.1038/srep06522 PMC4185378 25283467

[46] Li, B., Chen, X., Yang, J.-Y., Gao, S. & Bai, F. Intracellular ATP concentration is a key regulator of Bacterial Cell Fate. *Journal of Bacteriology* **206**, (2024). 10.1128/jb.00208-24 PMC11656805 39530704

[47] Byrd, A. K. & Raney, K. D. Superfamily 2 helicases. *Front Biosci. (Landmark Ed.)* **17**, 2070–2088 (2012). 1. 22652765 10.2741/4038 PMC3775597

[48] An, L. et al. Characterization of a Thermostable UvrD Helicase and Its Participation in Helicase-dependent Amplification*. *J. Biol. Chem.* **280**, 28952–28958 (2005). 15955821 10.1074/jbc.M503096200 PMC1361353

[49] Donlin, M. J., Patel, S. S. & Johnson, K. A. Kinetic partitioning between the exonuclease and polymerase sites in DNA error correction. *Biochemistry* **30**, 538–546 (1991). 1988042 10.1021/bi00216a031

[50] Liu, M.-S. et al. Engineered CRISPR/Cas9 enzymes improve discrimination by slowing DNA cleavage to allow release of off-target DNA. *Nature Communications* **11**, (2020). 10.1038/s41467-020-17411-1 PMC7367838 32681021

[51] Schwartz, E. A. et al. Structural rearrangements allow nucleic acid discrimination by type I-D cascade. *Nature Communications* **13**, (2022). 10.1038/s41467-022-30402-8 PMC9123187 35595728

[52] Groisman, E. A. et al. Bacterial Mg^2+^ homeostasis, transport, and virulence. *Annu. Rev. Genet.* **47**, 625–646 (2013). 24079267 10.1146/annurev-genet-051313-051025 PMC4059682

[53] Hernandez, A. J. & Richardson, C. C. Gp2.5, the Multifunctional Bacteriophage T7 Single-stranded DNA Binding Protein. *Semin Cell Dev. Biol.* **86**, 92–101 (2019). 29588157 10.1016/j.semcdb.2018.03.018 PMC6162179

[54] Hollis, T., Stattel, J. M., Walther, D. S., Richardson, C. C. & Ellenberger, T. Structure of the gene 2.5 protein, a single-stranded DNA binding protein encoded by bacteriophage T7. *Proc. Natl. Acad. Sci. USA*. **98**, 9557–9562 (2001). 1. 11481454 10.1073/pnas.171317698 PMC55491

[55] Zhang, H. et al. CTCF and R-loops are boundaries of cohesin-mediated DNA looping. *Mol. Cell* **83**, 2856–2871.e8 (2023). 37536339 10.1016/j.molcel.2023.07.006

[56] Soniat, M. M. et al. Chapter Eleven - Next-Generation DNA Curtains for Single-Molecule Studies of Homologous Recombination. in *Methods in Enzymology* (ed. Eichman, B. F.) **592** 259–281 (Academic Press, 2017). 10.1016/bs.mie.2017.03.011 PMC5564670 28668123

[57] Sami, A. A., Arabia, S., Sarker, R. H. & Islam, T. Deciphering the role of helicases and translocases: A multifunctional gene family safeguarding plants from diverse environmental adversities. *Curr. Plant Biol.* **26**, 100204 (2021).

[58] Shao, Q., Hawkins, A. & Zeng, L. Phage DNA Dynamics in Cells with Different Fates. *Biophys. J.* **108**, 2048–2060 (2015). 25902444 10.1016/j.bpj.2015.03.027 PMC4407255

[59] Yu, Z., Guan, J., Hanson, C., Duong, T. & Zeng, L. Fine-tuned spatiotemporal dynamics of DNA replication during phage lambda infection. *J. Virol.* **98**, e01128–24 (2024). 39480083 10.1128/jvi.01128-24 PMC11575281

[60] Zhao, L., Pellenz, S. & Stoddard, B. L. Activity and Specificity of the Bacterial PD-(D/E)XK Homing Endonuclease I-Ssp6803I. *J. Mol. Biol.* **385**, 1498–1510 (2009). 19038269 10.1016/j.jmb.2008.10.096 PMC3008403

[61] Horton, N. C., Newberry, K. J. & Perona, J. J. Metal ion-mediated substrate-assisted catalysis in type II restriction endonucleases. *Proc. Natl. Acad. Sci.* **95**, 13489–13494 (1998). 9811827 10.1073/pnas.95.23.13489 PMC24846

[62] Pingoud, A. & Jeltsch, A. Structure and function of type II restriction endonucleases. *Nucleic Acids Res*. **29**, 3705–3727 (2001). 11557805 10.1093/nar/29.18.3705 PMC55916

[63] Pingoud, V. et al. On the Divalent Metal Ion Dependence of DNA Cleavage by Restriction Endonucleases of the EcoRI Family. *J. Mol. Biol.* **393**, 140–160 (2009). 19682999 10.1016/j.jmb.2009.08.011

