# Supplementary material for: Phage-encoded factor stimulates DNA degradation by the Hna anti-phage defense system
Source: Nat Commun. 2026 May 18;17:6544. doi: 10.1038/s41467-026-73157-2 (PMC13381803; doi:10.1038/s41467-026-73157-2)
Supplement: Supplementary file 1 — Supplementary Information [file 41467_2026_73157_MOESM1_ESM.pdf]

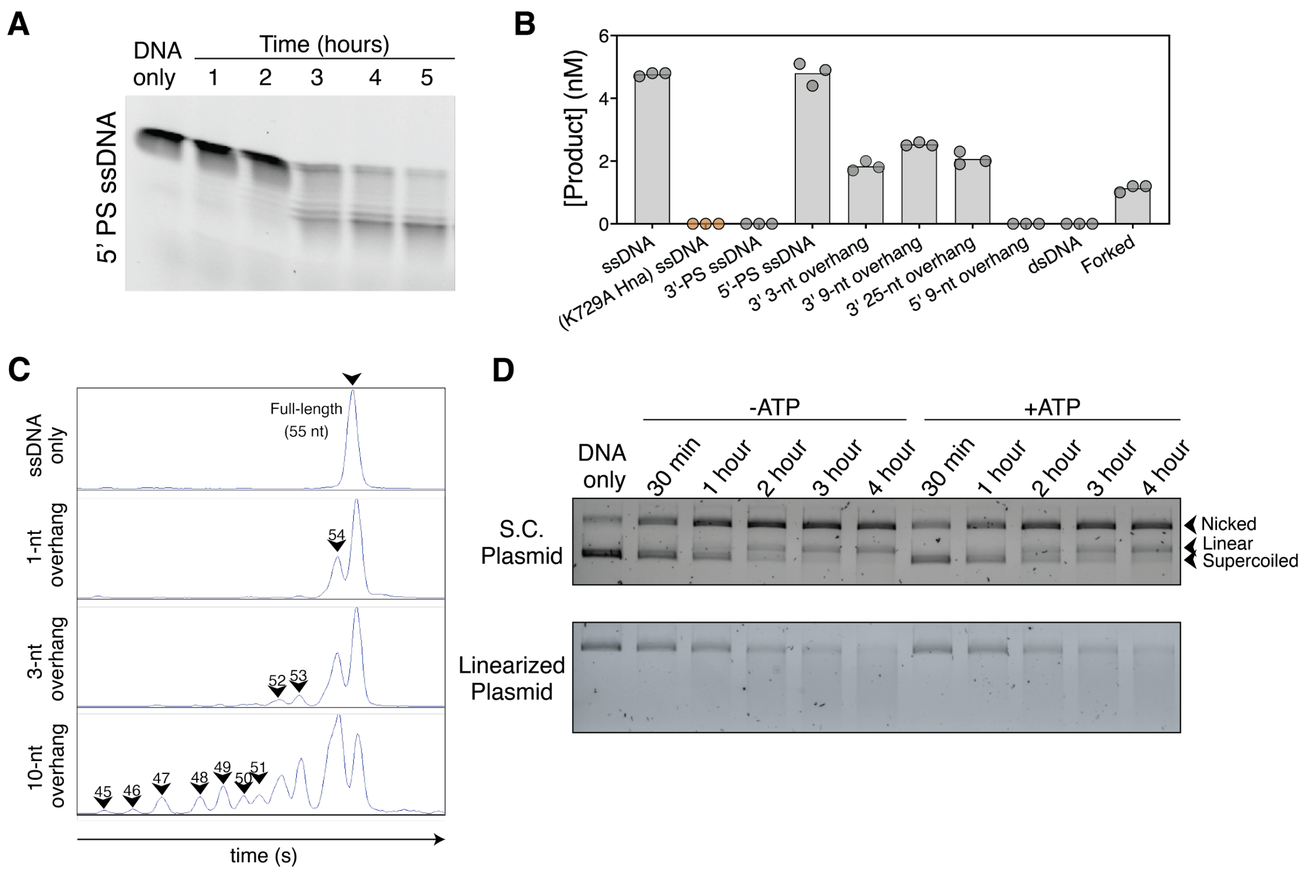

Supplementary Fig. 1 | Hna degrades single-stranded and plasmid DNA. A, Exonuclease activity over time using ssDNA with a series of 5'-phosphorothioate (PS) modifications showing unperturbed cleavage. Gel is representative of 3 independent experiments. **B,** Quantification of DNA cleavage by Hna in the presence of various substrates using capillary electrophoresis. Data represents product formation across three independent, biological replicates. **C,** Raw fluorescence peaks corresponding to differently sized fluorescently-labeled cleavage products. DNA only negative control (55 nucleotides) shown at the top. **D,** Time course Hna cleavage using supercoiled (S.C.) and linearized plasmid in the presence or absence of ATP. Distinct species of DNA are denoted on the right. Gel is representative of 4 independent experiments. Source data are provided as a Source Data file.

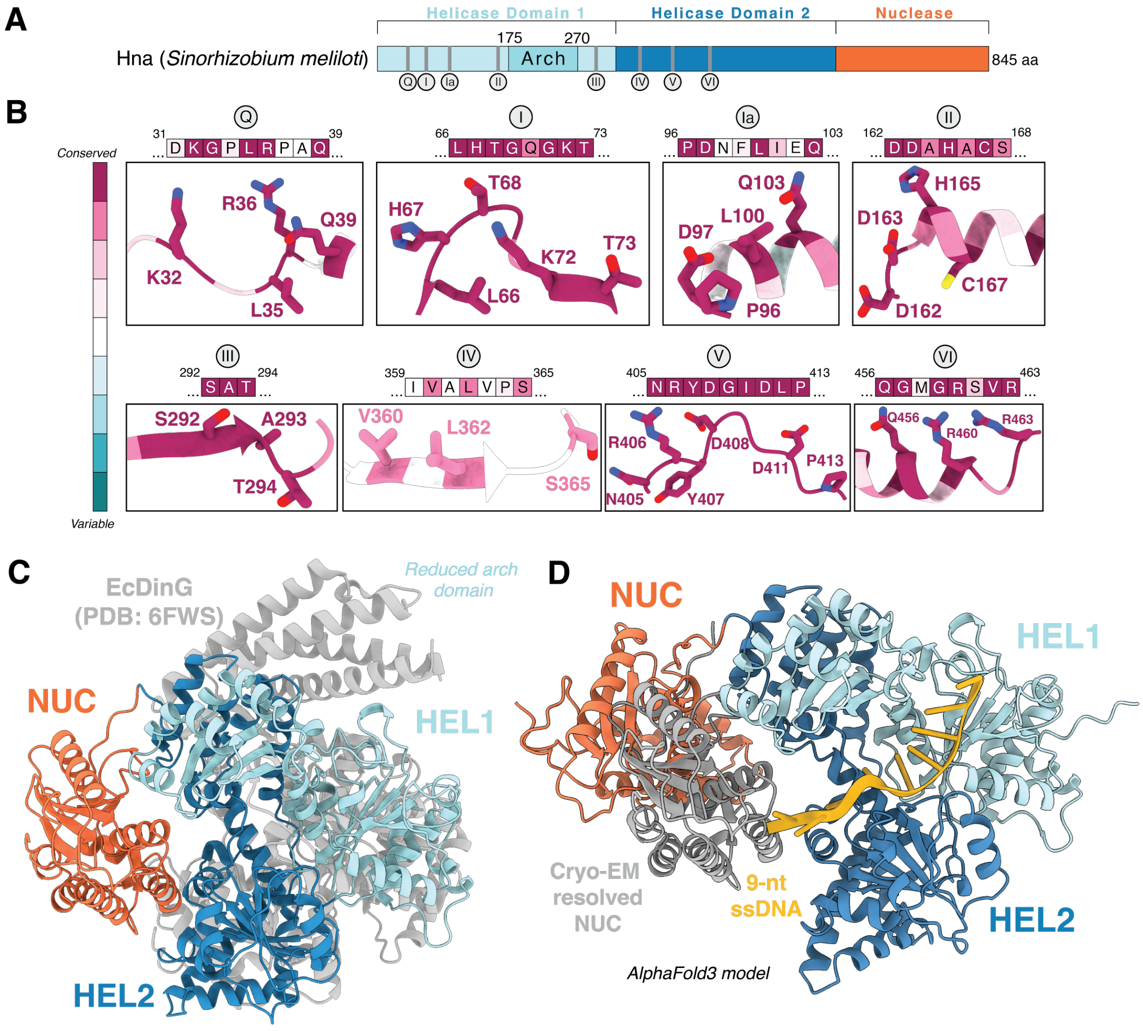

Supplementary Fig. 2 | Hna structural motifs and conservation. A, Domain organization of Hna from *S. meliloti*. The two helicase modules and C-terminal nuclease domain are shown in blue and orange, respectively. Canonical HEL1 and HEL2 functional motifs are labeled, as well as the HEL1 Arch domain. **B,** Magnified view of each identified SF2 helicase motif. Residues are colored by conservation with position and sequence listed above. **C,** Overlay of determined Hna monomer structure (colored) and structurally similar protein, *EcDinG* (PDB: 6FWS; gray). Hna and DinG helicase domains are highly similar with notably reduced Arch domain in Hna. **D,** AlphaFold3 prediction of Hna bound to a 9-nucleotide ssDNA substrate (colored) overlaid with the NUC domain of Hna monomer structure (gray). The predicted substrate fully occupies the Hna DNA binding site and promotes a unique NUC domain rearrangement.

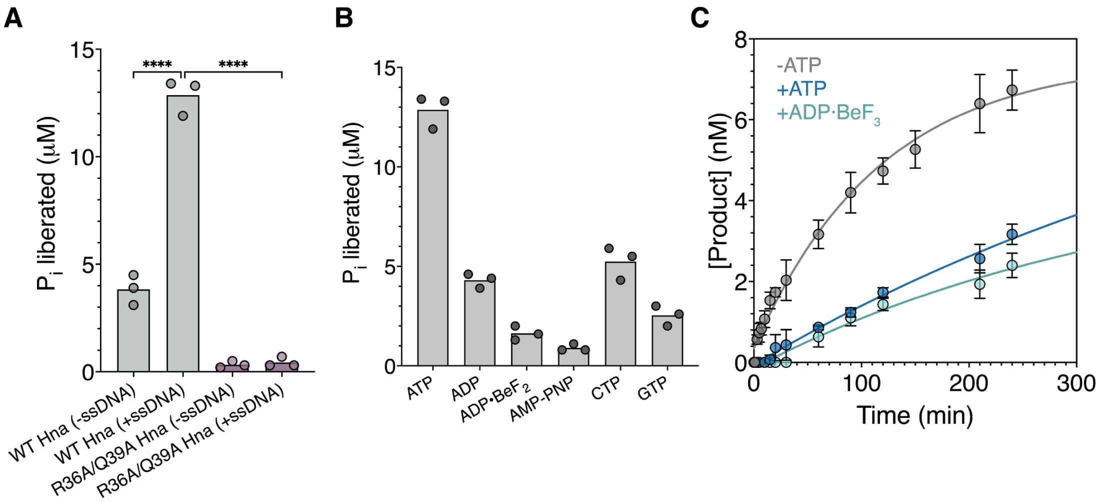

Supplementary Fig. 3 | Hna exhibits ssDNA-stimulated ATPase activity. A, Malachite green ATPase assay using wild-type Hna or ATP-binding mutant (R36A/Q39A) with and without ssDNA present (n=3 independent, biological replicates). Phosphate concentration was determined after 30 minutes of incubation. Statistical significance is evaluated via one-way ANOVA; $*p < 0.05$, $**p < 0.01$, $***p < 0.001$. **B,** Malachite green phosphate detection assay using wild-type Hna with ssDNA present and various nucleotide analogs. Phosphate concentration was determined after 30 minutes of incubation (n=3 independent, biological replicates). **C,** Quantification of ssDNA cleavage by Hna in the presence and absence of ATP or ADP·BeF_3 using capillary electrophoresis. Data shown are the mean ± standard deviation of three independent experiments for each condition. Source data are provided as a Source Data file.

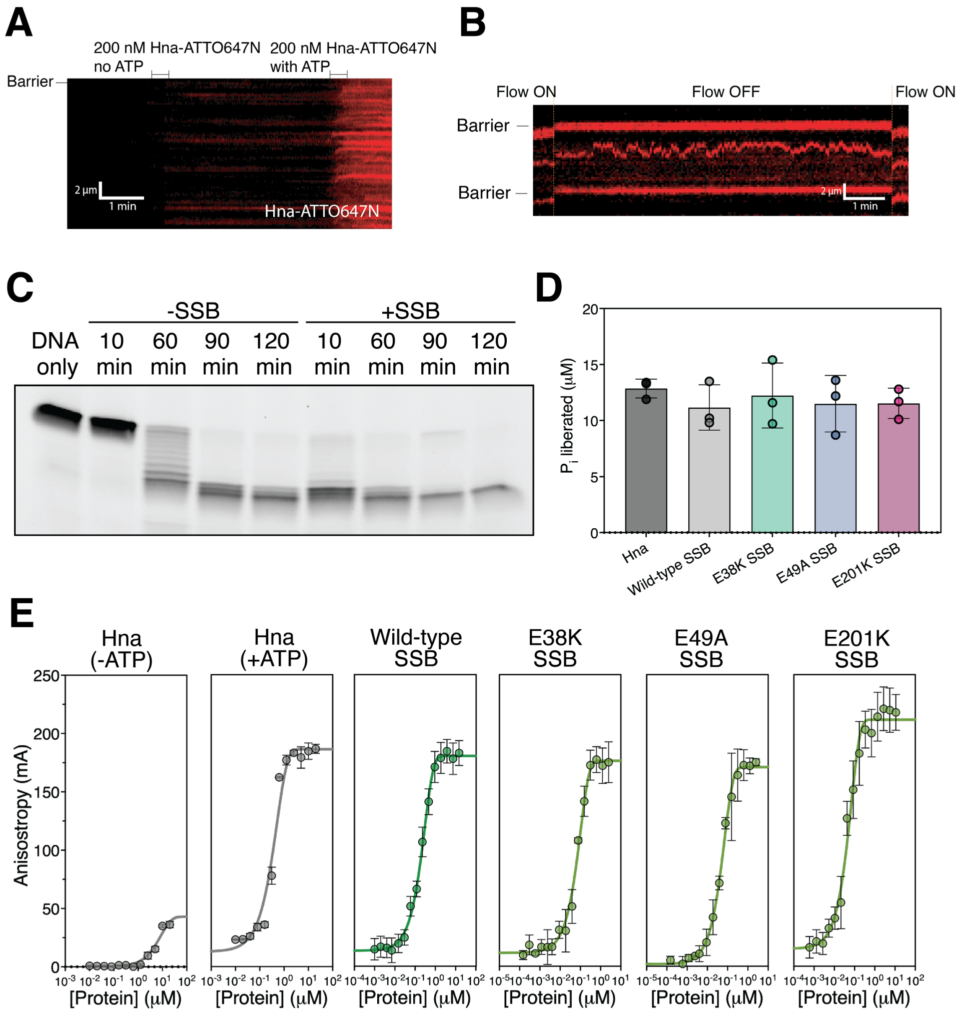

Supplementary Fig. 4 | Hna is an ATP-dependent DNA binding protein. A, Representative kymograph showing ATP-dependent binding of ssDNA by Hna (red). ATP was injected at the time indicated on the kymograph promoting increased binding events. **B,** Representative kymograph showing passive diffusion of Hna (red) along ssDNA in the absence of buffer flow. **C,** Time course cleavage of ssDNA by Hna in the absence or presence of phage-encoded 5A SSB. **D,** Malachite green ATPase assay using wild-type Hna only (Hna) or combined with various SSB mutants with single-stranded DNA present. Phosphate concentration was determined after 30 minutes of incubation 37°C. Data shown are the mean ± standard deviation of three independent, biological replicates for each condition. **E,** Hna and 5A SSB binding curves after incubation with fluorescently labeled ssDNA. Data shown are the mean ± standard deviation of three independent, biological replicates fit to a one-phase decay function. Source data are provided as a Source Data file.

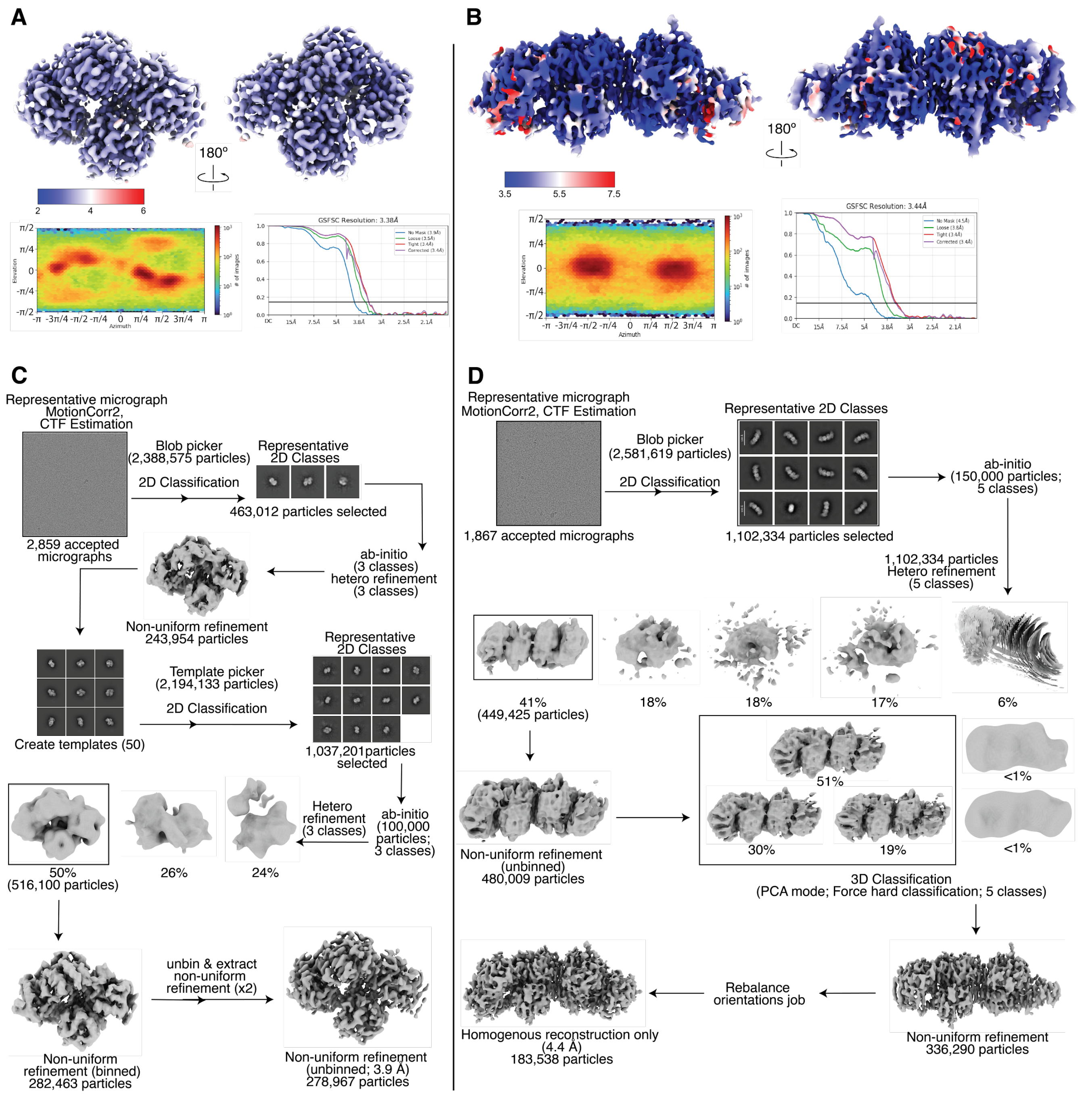

Supplementary Fig. 5 | Structural analysis of Hna. A, Unsharpened maps colored by local resolution (top) for Hna monomer and **B,** Hna dimer. Accompanying gold-standard FSC curves for cryo-EM reconstructions (left) with Euler diagrams showing orientation distributions of cryo-EM reconstructions (right). Resolutions were estimated at FSC=0.143. **C,** Data processing pipelines for Hna monomer and **D,** Hna dimer.

Supplementary Table 1 | List of DNA sequences used in this study.

**Oligo Name**	**Sequence (5’ to 3’)**
ssDNA (55-nt)	agctgacgtttgtatgtctgcgtctcatctttatgcgtcagcagagatttctgct
dsDNA complement	agcagaaatctctgctgacgcataaagatgagacgcagacatacaaacgtcagct
3’ 1-base overhang complement	gcagaaatctctgctgacgcataaagatgagacgcagacatacaaacgtcagct
3’ 3-base overhang complement	agaaatctctgctgacgcataaagatgagacgcagacatacaaacgtcagct
3’ 9-base overhang complement	ctctgctgacgcataaagatgagacgcagacatacaaacgtcagct
3’ 10-base overhang complement	tctgctgacgcataaagatgagacgcagacatacaaacgtcagct
3’ 25-base overhang complement	agatgagacgcagacatacaaacgtcagct
5’ 9-base overhang complement	agcagaaatctctgctgacgcataaagatgagacgcagacatacaa
Forked complement	tgagacgcagacatacaaacgtcagcttgagacgcagacatacaaacgtcagct

Supplementary Table 2 | Cryo-EM data collection, refinement, and validations statistics.

	Hna Monomer (EMD-75470; PDB 10UJ)	Hna Dimer (EMD-73047; PDB 9YKJ)
**Data Collection and Processing**		
Voltage (kV)	200	200
Electron exposure (e-/Å^2)	49	49
Defocus range (μm)	-1.5 to -2.5	-1.5 to -2.5
Pixel size (Å)	0.94	0.94
Symmetry imposed	C1	C1
Initial particle images (no.)	2,194,133	2,581,619
Final particle images (no.)	278,967	185,538
Map resolution (Å)	3.9	4.4
FSC threshold	0.143	0.143
**Refinement**		
Initial model used (PDB code)	AlphaFold3	AlphaFold3
Model resolution (Å)	3.8	3.7
FSC threshold	0.5	0.5
Map sharpening B factor (Å^2)	167.3	121.9
Model composition		
Non-hydrogen atoms	6490	5952
Protein residues	821	1205
Nucleotides	0	0
R.m.s deviations		
Bond lengths (Å)	0.004	0.003
Bond angles (°)	0.808	0.819
Validation		
MolProbity score	1.2	1.18
Clashscore	3.93	0.57
Poor rotamers (%)	0	0
Ramachandran plot		
Favored (%)	97.92	92.03
Allowed (%)	2.08	7.29
Disallowed (%)	0	0
