# Supplementary material for: Phage-encoded factor stimulates DNA degradation by the Hna anti-phage defense system
Source: Nat Commun. 2026 May 18;17:6544. doi: 10.1038/s41467-026-73157-2 (PMC13381803; doi:10.1038/s41467-026-73157-2)
Supplement: Supplementary file 2 — Reporting Summary [file 41467_2026_73157_MOESM2_ESM.pdf]

Corresponding author(s): David W. Taylor
Last updated by author(s): Apr 14, 2026

# Reporting Summary

Nature Portfolio wishes to improve the reproducibility of the work that we publish. This form provides structure for consistency and transparency in reporting. For further information on Nature Portfolio policies, see our Editorial Policies and the Editorial Policy Checklist.

## Statistics

For all statistical analyses, confirm that the following items are present in the figure legend, table legend, main text, or Methods section.

| n/a | Confirmed | |
|---|---|---|
| ☐ | ☒ | The exact sample size ($n$) for each experimental group/condition, given as a discrete number and unit of measurement |
| ☐ | ☒ | A statement on whether measurements were taken from distinct samples or whether the same sample was measured repeatedly |
| ☐ | ☒ | The statistical test(s) used AND whether they are one- or two-sided<br>*Only common tests should be described solely by name; describe more complex techniques in the Methods section.* |
| ☒ | ☐ | A description of all covariates tested |
| ☒ | ☐ | A description of any assumptions or corrections, such as tests of normality and adjustment for multiple comparisons |
| ☐ | ☒ | A full description of the statistical parameters including central tendency (e.g. means) or other basic estimates (e.g. regression coefficient) AND variation (e.g. standard deviation) or associated estimates of uncertainty (e.g. confidence intervals) |
| ☐ | ☒ | For null hypothesis testing, the test statistic (e.g. $F$, $t$, $r$) with confidence intervals, effect sizes, degrees of freedom and $P$ value noted<br>*Give P values as exact values whenever suitable.* |
| ☒ | ☐ | For Bayesian analysis, information on the choice of priors and Markov chain Monte Carlo settings |
| ☒ | ☐ | For hierarchical and complex designs, identification of the appropriate level for tests and full reporting of outcomes |
| ☒ | ☐ | Estimates of effect sizes (e.g. Cohen's $d$, Pearson's $r$), indicating how they were calculated |

*Our web collection on statistics for biologists contains articles on many of the points above.*

## Software and code

Policy information about availability of computer code

| | |
|---|---|
| Data collection | For both structures, data was collected using a FEI Glacios cryo-EM microscope (200 kV) equipped with a Falcon 4 direct electron detector (Gatan). Movies were recorded wiith a pixel size of 0.94 Å and a total exposure time of 15 seconds for an accumulated dose of 49 e-/Å2. Motion correction, contrast transfer function (CTF) estimation and particle picking were carried out in cryoSPARC live v4.0. |
| Data analysis | Data processing was performed in cryoSPARC v.4.4. Both Hna structures were initially rigid body fit using models generated from AlphaFold3 predictions within ChimeraX. For the Hna monomer, domain conformations were individually rigid body fit, then further refined through interactive rounds of modeling in Isolde (version 1.10) and Coot (version 0.9.8.95). The model was subsequently processed using real space refinement in Phenix (version 1.21) to generate final validation scores. Detailed structural analysis pipeline and validation statistics for both structures are provided. |

For manuscripts utilizing custom algorithms or software that are central to the research but not yet described in published literature, software must be made available to editors and reviewers. We strongly encourage code deposition in a community repository (e.g. GitHub). See the Nature Portfolio guidelines for submitting code & software for further information.

## Data

Policy information about availability of data

All manuscripts must include a data availability statement. This statement should provide the following information, where applicable:

- Accession codes, unique identifiers, or web links for publicly available datasets
- A description of any restrictions on data availability
- For clinical datasets or third party data, please ensure that the statement adheres to our policy

The structures and associated atomic coordinates have been deposited into the Electron Microscopy Data Bank (EMDB) and Protein Data Bank (PDB) with accession codes: Hna Monomer (EMD-75470 [https://www.ebi.ac.uk/emdb/EMD-75470] and PDB 10UJ [http://doi.org/10.2210/pdb10UJ/pdb]) and Hna Dimer (EMD-73047 [https://www.ebi.ac.uk/emdb/EMD-73047] and PDB 9YKJ [http://doi.org/10.2210/pdb9YKJ/pdb]). Source data are provided with this paper.

## Research involving human participants, their data, or biological material

Policy information about studies with human participants or human data. See also policy information about sex, gender (identity/presentation), and sexual orientation and race, ethnicity and racism.

| | |
|---|---|
| Reporting on sex and gender | Not applicable. |
| Reporting on race, ethnicity, or other socially relevant groupings | Not applicable. |
| Population characteristics | Not applicable. |
| Recruitment | Not applicable. |
| Ethics oversight | Not applicable. |

Note that full information on the approval of the study protocol must also be provided in the manuscript.

# Field-specific reporting

Please select the one below that is the best fit for your research. If you are not sure, read the appropriate sections before making your selection.

☒ Life sciences ☐ Behavioural & social sciences ☐ Ecological, evolutionary & environmental sciences

For a reference copy of the document with all sections, see nature.com/documents/nr-reporting-summary-flat.pdf

# Life sciences study design

All studies must disclose on these points even when the disclosure is negative.

| | |
|---|---|
| Sample size | Sample sizes were determined by the number of samples required to derive statistical measurements. |
| Data exclusions | No data was excluded. |
| Replication | All experiments were replicated at least three times with similar results. |
| Randomization | Particle orientations are randomized during processing. |
| Blinding | No blinding was performed. The experiments were observational and not subject to bias. |

# Reporting for specific materials, systems and methods

We require information from authors about some types of materials, experimental systems and methods used in many studies. Here, indicate whether each material, system or method listed is relevant to your study. If you are not sure if a list item applies to your research, read the appropriate section before selecting a response.

## Materials & experimental systems

| n/a | Involved in the study |
|---|---|
| ☐ | ☒ Antibodies |
| ☒ | ☐ Eukaryotic cell lines |
| ☒ | ☐ Palaeontology and archaeology |
| ☒ | ☐ Animals and other organisms |
| ☒ | ☐ Clinical data |
| ☒ | ☐ Dual use research of concern |
| ☒ | ☐ Plants |

## Methods

| n/a | Involved in the study |
|---|---|
| ☒ | ☐ ChIP-seq |
| ☒ | ☐ Flow cytometry |
| ☒ | ☐ MRI-based neuroimaging |

# Antibodies

Antibodies used: anti-FLAG (200 nm) and anti-Mouse-IgG-ATTO647N (200 nM)

Validation: Detects a single band of protein on a Western Blot from mammalian crude cell lysates by chemiluminescent probing.

# Plants

Seed stocks: Not applicable.

Novel plant genotypes: Not applicable.

Authentication: Not applicable.
