# Supplementary material for: Phage-encoded factor stimulates DNA degradation by the Hna anti-phage defense system
Source: Nat Commun. 2026 May 18;17:6544. doi: 10.1038/s41467-026-73157-2 (PMC13381803; doi:10.1038/s41467-026-73157-2)
Supplement: Supplementary file 3 — Transparent Peer Review file [file 41467_2026_73157_MOESM3_ESM.pdf]

nature portfolio

Peer Review File

Phage-encoded factor stimulates DNA degradation by the Hna anti-phage defense system

Corresponding Author: Dr David Taylor

This file contains all reviewer reports in order by version, followed by all author rebuttals in order by version.

Version 0:

Reviewer comments:

Reviewer #1

(Remarks to the Author)
Hna is a widely distributed bacterial anti-phage defense system that provides resistance to various phages through an abortive infection response. However, its mechanism of action remains elusive. In this study, Hooper et al. reveal that Hna functions as a 3'–5' single-stranded DNA exonuclease and exists as an auto-inhibited dimer under physiological ATP concentrations. Furthermore, the authors demonstrate that a phage-encoded single-stranded DNA-binding protein binds to Hna, facilitating the dissociation of the Hna dimer and consequently activating its nuclease activity. Overall, this study elucidates the molecular mechanisms underlying Hna-mediated anti-phage activity and enhances our understanding of diverse bacterial anti-phage defense systems. However, the authors should consider the following points to improve the manuscript prior to publication.

Points:

Hna is a DNA exonuclease:
It would be informative if the authors could provide more details about the structure of the NUC domain (e.g., its active-site structure and a comparison with structurally similar proteins). Do typical PD-(D/E)XK nucleases function as 3'–5' DNA exonucleases?

ATP inhibits Hna nuclease activity:
The authors examined Hna nuclease activity at ATP concentrations ranging from 0 to 1 mM and found approximately a 10-fold reduction in ssDNA cleavage in the presence of 1 mM ATP compared to conditions without ATP after 1 hour of incubation (Extended Data Fig. 3D). Furthermore, the authors determined the cryo-EM structure of Hna bound to ATP analogs (ADP-BeF3) and discovered that Hna forms an autoinhibitory dimer in the presence of ATP. Based on these biochemical and structural data, the authors concluded that ATP functions as a negative regulator under physiological ATP concentrations, suppressing Hna nuclease activity and protecting bacterial cells prior to phage infection. This model is intriguing but requires further clarification.

It would be helpful to provide information about physiological ATP concentrations. Does 1 mM ATP represent a physiological concentration? Additionally, it could be better to test higher ATP concentrations as well?

Unlike in the Hna monomeric structure, in its dimeric structure, the NUC domains were less resolved in the cryo-EM density map, indicating increased flexibility in the domains upon ATP-dependent dimerization. Accordingly, the authors proposed that these conformational differences explain the auto-inhibition of Hna exonuclease activity through dimerization. However, the following points should be clarified.

It is unclear why the flexibility of the NUC domains increases upon dimerization. Do conformational changes in the HEL1/HEL2 domains result in different NUC-HEL1/HEL2 interactions, thereby increasing NUC flexibility? The authors should provide figures showing comparisons between the monomer and dimer (protomers A and B) regarding the overall structures and NUC-HEL1/HEL2 interfaces, as well as comparisons between protomer A and protomer B from the same angle (including superimposition if it is informative).

Why is the NUC domain in protomer A partially disordered while that in protomer B is fully disordered in the dimer structure?

The most important question is why Hna exonuclease activity is inhibited upon dimerization (i.e., enhancement of the flexibility of the NUC domain), as the NUC active sites appear to be located outside rather than inside the dimer. This should be clarified.

Does the helicase domain participate in ssDNA binding? AF3-predicted models of the Hna monomer and dimer bound to ssDNA might provide additional insights into the recognition and cleavage of ssDNA substrates.

Fig. 1A-C: ATP concentrations should be specified in the legend, as they significantly affect the nuclease activity of Hna.

Figs. 2A and 3A: How can the authors determine if the shifted bands correspond to the Hna dimer rather than higher-order oligomeric states, even though the cryo-EM structure indicates that Hna forms a dimer in the presence of ATP? It would be better to examine oligomeric states using other techniques, such as SEC, in addition to EMSA.

Fig. 3C: It would be informative to show the positions of phage escape mutations in the structure.

Fig. 3H: Since these data are important to support the authors' model, gel data should also be presented either in the main or supplementary figure.

Since Extended Data Fig. 3D (ATP dependency on Hna exonuclease activity) is important to support the authors' claim, it should be moved to Fig. 1.

Minor points:

Fig. 1A, B, etc.: A DNA size marker should be run alongside the samples and clearly labeled.

Fig. 1F, H, etc.: It would be beneficial to revise the color scheme to differentiate between the various conditions.

Fig. 2B: The two bound ADP molecules should be labeled, or an explanation should be included in the legend. Additionally, it would be helpful to indicate the likely location of the disordered NUC domain in protomer B with a dashed circle.

Extended Data Figs. 3B, C, and 4: Some labels are too small (less than 5 pt) to be discernible. These figures should be revised to enhance readability.

Reviewer #2

(Remarks to the Author)
This manuscript investigates the molecular mechanism of the Hna anti-phage defence system from Sinorhizobium meliloti. The authors show that Hna acts as a 3′→5′ single-stranded DNA exonuclease whose activity is strongly inhibited by ATP-dependent homodimerisation. Using cryo-EM, they resolve both monomeric and dimeric conformations and propose that dimerisation disrupts the nuclease domain's ability to engage DNA. They further report that a phage-encoded single-stranded DNA–binding protein (5A SSB) activates Hna by breaking this autoinhibited state, leading to robust exonuclease activity even under inhibitory ATP concentrations. Single-molecule assays demonstrate ATP-dependent localisation of Hna to ssDNA, and biochemical assays show that wild-type phage SSB stimulates Hna, whereas phage escape mutants (with increased DNA affinity or altered oligomerisation) fail to do so. The authors propose a model in which Hna remains silent under physiological conditions but becomes hyperactive during phage infection when SSB and ssDNA accumulate, triggering abortive infection.

Major concerns
1. The mechanistic model is entirely in vitro and untested in vivo.
Although prior work showed that 5A SSB can trigger Hna-dependent abortive infection, this manuscript does not test whether any of the newly proposed mechanistic steps occur in cells. Specifically, ATP-dependent autoinhibitory dimerisation, SSB-driven dimer disruption, and the behaviour of escape mutants are never validated in a biological system. There are no infection assays, toxicity assays, or cellular readouts using the mutants they generated. This leaves a major gap between the biochemical mechanism and physiological relevance.

2. The central Hna–SSB interaction is not rigorously demonstrated.
The heterodimer model is built predominantly from AlphaFold3 predictions. Direct biochemical proof of interaction is limited to a qualitative native gel showing a shifted species. The single-molecule DNA-curtain experiments ,which should be the clearest assay , show almost no consistent colocalisation between GFP–SSB and Hna. Without quantitative binding assays (ITC, BLI, MST) or clear single-molecule co-detection(fluorescence microscopy or even a pulldown), the central conclusion that SSB physically disrupts the Hna dimer remains under-supported.

3. No DNA-bound Hna structure, leaving substrate-binding and catalytic engagement speculative.
The authors never observe DNA density in their cryo-EM structures. Yet the model depends on precise hypotheses about DNA engagement, transient contacts, and NUC-domain positioning. Without structural evidence for even a minimal DNA footprint, the mechanistic interpretation, and the claim that Hna has a "shallow" binding mode, remains speculative. While I understand the difficulties of the technique, it'd be better of the speculative nature of some of the conclusions was more

clearly stated.

4. The nuclease domain is unresolved in the dimer, but the inhibition model depends entirely on its displacement.
Large portions of the NUC domain are missing from the EM density in the dimer structure, yet the authors attribute ATP-dependent inhibition to NUC-domain misalignment and flexibility. This is inferred, not shown. Without a resolved NUC-domain conformation in the inhibited dimer, the structural basis of inhibition is incomplete. Again, I recognise the EM challenges, but a more clearly stated “limitation of the study” or speculative nature of some of the conclusions would be appreciated.

5. Escape mutant analysis is not mechanistically convincing.
The authors observe higher-order oligomers or higher DNA affinity in some mutants and infer this explains escape. But they never test:
– whether these mutants fail to bind Hna,
– whether they fail to disrupt the Hna dimer,
– whether they fail to stimulate nuclease activity,
– or whether the same effects occur in vivo.
The mechanistic interpretation is therefore correlative.

6. Several results that anchor major conclusions (EMSAs, gel shifts, nuclease gels) are qualitative. Band intensities, ratios of monomer/dimer, and kinetics of nuclease activity are not quantified. For a mechanistic paper, this weakens the robustness of the claims.

7. ATP inhibition mechanism is underdeveloped.
While the authors show ATP inhibits nuclease activity, they do not evaluate:
– ATPase kinetics under physiological ATP/ADP ratios,
– the equilibrium between monomer and dimer across ATP concentrations,
– whether ATP hydrolysis meaningfully contributes to dimer dissolution,
– or whether SSB influences ATPase dynamics.
Thus, the inhibitory mechanism lacks depth.

8. Ambiguity in single-molecule conclusions.
While the DNA-curtain experiments confirm that Hna binds and diffuses on ssDNA, they do not reveal any SSB-dependent changes in Hna behaviour. Hna does not show increased binding, altered diffusion, or enhanced processivity upon addition of 5A SSB. More importantly, co-localisation between Hna and SSB on ssDNA is extremely rare, meaning the experiments never directly visualise the proposed SSB–Hna heterodimer or any SSB-triggered dissociation of the autoinhibited Hna dimer. As a result, the single-molecule observations fall short of demonstrating the central mechanistic claim that SSB physically engages and activates Hna.
9. The Discussion exaggerates the novelty of SSB-triggered activation.
The text repeatedly frames this mechanism as unusual, although similar SSB-dependent activation exists in Hachiman, AbpAB, Nhi, and others. A more measured comparison to known precedents would improve accuracy and framing.

Minor concerns:
1. Reporting of single-molecule assay conditions is incomplete or inconsistent.
Key experimental parameters (protein concentration, injection concentration, flow rate, dwell times, surface density, and imaging buffer composition) are scattered through the Methods. This makes it difficult to assess reproducibility or the sensitivity of the observations.
2. ATP concentrations differ across experiments without rationale or discussion.
Some assays use 20 µM ATP (ATPase assays), others use 1 mM ATP (nuclease assays, EMSAs, DNA curtains). The physiological relevance of these concentrations is not addressed, nor is it explained why different ATP levels are used for different readouts.
3. Figure presentation needs clearer lane and marker annotations.
Several gel-based figures (especially native gels and cleavage assays) lack molecular weight markers, clear sample labels, or schematics of which lanes contain which components. This makes interpretation harder than necessary.
4. No quantitative or structural analysis of NUC-domain flexibility.
The nuclease domain is unresolved in the dimer structure, yet the inhibition model relies on its displacement. The manuscript provides no quantification (e.g., local resolution, variance maps) to support claims about its flexibility.
5. The comparison between monomer and dimer cryo-EM maps is insufficiently explicit.
While the text mentions conformational rearrangements, the figures do not include direct overlays or domain-specific RMSD comparisons that would clarify how ATP-binding drives inhibition.

Reviewer #3

(Remarks to the Author)
The manuscript “Phage-encoded factor stimulates DNA degradation by the Hna anti-phage defense system” summitted by

Hooper et al. uses a combination of biophysical and biochemical measurements to describe the molecular mechanism of the Hna anti-phage immune system encoded by S. meliloti. My role as a reviewer is limited to assessment of the technical aspects of the cryo-EM work so I will comment only on that. Unfortunately, I do not find the maps of the Hna complex very compelling – visual inspection suggests the resolutions are substantially worse than the reported 3.3 Å for the dimer and 3.4 Å for the monomer. Indeed, the PDB validation reports (which should have been submitted with the manuscript for review) estimate the unmasked resolutions to be 4.49 Å (dimer) and 3.95 Å (monomer), which look more appropriate. Furthermore, the maps appear to be aggressively sharpened and the sharpening process is not described in the materials and methods.

Due to the low resolution of the maps, the protein models are also not convincing except as general footprints showing the relative placement of protein domains. Rigid body fitting of domains from Alphafold3 predictions is appropriate into this type of map, but I am not convinced that refinements with Coot, Isolde, and Phenix are meaningful. Especially worrisome is the placement of ligands like calcium ions and ADP – these are not supported by the data. The poor map-model fits are reflected in the low atom inclusion scores and Q-scores present in the PDB validation reports.

Overall, this cryo-EM work is not up to par for a key piece of data in this manuscript. If the manuscript were re-written to honestly communicate the limitations of the maps and models I would reconsider. But as written, the manuscript is misleading as to the quality of the structural biology work and I must therefore decline to recommend it for publication. I know high resolution maps are very challenging to achieve for small molecules, and I commend the authors for what they were able to accomplish – but I believe there must be more transparency in the description of the resulting data.

Version 1:

Reviewer comments:

Reviewer #1

(Remarks to the Author)
The authors have addressed my previous concerns. Although the resolutions of the cryo-EM structures—particularly the Hna dimer—are limited, I find that the proposed mechanism of Hna action is supported by biochemical data together with the AlphaFold3-predicted model.

Reviewer #2

(Remarks to the Author)
I am satisfied that the authors have addressed my comments thoroughly

Reviewer #3

(Remarks to the Author)
The revised manuscript submitted by Hooper et al. has addressed my greatest concerns with the updated resolution estimates for the cryo-EM maps and constraints on how the dimer model was built/described (namely limiting it to rigid body fitting of domains from AF3 predictions rather than refining with Coot/Phenix/Isolde). The revised text remains interesting and compelling but now accurately communicates the limitations of the structural work while still supporting the biochemistry with the structural findings.

Additionally, my apologies for the accusation of aggressive sharpening. I realize now that I was misinterpreting the effects of preferred orientations for the dimer, and I see now that you commented on this in the methods section and in Extended Figure 5. To complement these workflow descriptions, I would suggest adding a “top view” and/or “bottom view” of the local resolution maps in that figure to communicate the issue visually. I think this would help readers who look at the map to understand why it looks so low resolution from one angle.

Kudos on a nice paper.

Open Access This Peer Review File is licensed under a Creative Commons Attribution 4.0 International License, which permits use, sharing, adaptation, distribution and reproduction in any medium or format, as long as you give appropriate credit to the original author(s) and the source, provide a link to the Creative Commons license, and indicate if changes were made.
In cases where reviewers are anonymous, credit should be given to 'Anonymous Referee' and the source.
The images or other third party material in this Peer Review File are included in the article's Creative Commons license, unless indicated otherwise in a credit line to the material. If material is not included in the article's Creative Commons license and your intended use is not permitted by statutory regulation or exceeds the permitted use, you will need to obtain permission directly from the copyright holder.
To view a copy of this license, visit https://creativecommons.org/licenses/by/4.0/

REVIEWER #1

1. Hna is a DNA exonuclease: It would be informative if the authors could provide more details about the structure of the NUC domain (e.g., its active-site structure and a comparison with structurally similar proteins). Do typical PD-(D/E)XK nucleases function as 3'–5' DNA exonucleases?

We thank the reviewer for this suggestion. We agree that a more detailed description of the Hna NUC domain would provide better clarity to the role and organization of the Hna NUC domain and how it relates to other well characterized PD-(D/E)XK nucleases.

To address this shortcoming, we have improved our depiction of the Hna NUC domain active site, including its highly conserved αβββαβ configuration (**Figure 1E**). Additionally, we have provided direct structural comparisons between Hna and the structurally similar PD-(D/E)XK nucleases, EcDinG (**Extended Figure 2C**). The following text has also been added to emphasize the structure and functional similarities to DinG proteins:

"Structural analysis revealed that Hna resembles a subclass of DinG-like proteins, termed ExoDinG, that also function as 3'—5' ssDNA exonucleases. Comparison of Hna with structurally similar members of the DinG and XPD protein families revealed that Hna retains characteristic sequence motifs associated with ATP binding and hydrolysis in SF2 proteins, as well as a reduced Arch domain, yet lacks the canonical iron-sulfur cluster[35, 37, 38, 39, 40, 49]."

2. ATP inhibits Hna nuclease activity: The authors examined Hna nuclease activity at ATP concentrations ranging from 0 to 1 mM and found approximately a 10-fold reduction in ssDNA cleavage in the presence of 1 mM ATP compared to conditions without ATP after 1 hour of incubation (Extended Data Fig. 3D). Furthermore, the authors determined the cryo-EM structure of Hna bound to ATP analogs (ADP-BeF3) and discovered that Hna forms an autoinhibitory dimer in the presence of ATP. Based on these biochemical and structural data, the authors concluded that ATP functions as a negative regulator under physiological ATP concentrations, suppressing Hna nuclease activity and protecting bacterial cells prior to phage infection. This model is intriguing but requires further clarification. It would be helpful to provide information about physiological ATP concentrations. Does 1 mM ATP represent a physiological concentration? Additionally, it could be better to test higher ATP concentrations as well?

We agree with the reviewer that more extensive characterization of the ATP-mediated inhibition of Hna nuclease activity would strengthen the model presented in this study. To accommodate this request, we have performed additional DNA cleavage experiments with an expanded range of ATP concentrations (**Figure 1H**). Additionally, we have clarified that the ATP concentrations used in this study are consistent with intracellular concentrations of ATP recorded in comparable gram-negative bacteria with the following text:

"Additionally, substitution of ATP for the non-hydrolyzable analog, ADP·BeF3, yielded comparable inhibition of DNA degradation, suggesting that Hna enters an inhibited state upon ATP binding rather than following ATP hydrolysis (Extended Fig. 3C)[43, 44, 45]. These data suggest that ATP acts as a negative regulator of Hna nuclease activity with considerable sensitivity at even sub-physiological levels of ATP (<1 mM)[46, 47]."

3. Unlike in the Hna monomeric structure, in its dimeric structure, the NUC domains were less resolved in the cryo-EM density map, indicating increased flexibility in the domains upon ATP-dependent

dimerization. Accordingly, the authors proposed that these conformational differences explain the auto-inhibition of Hna exonuclease activity through dimerization. However, the following points should be clarified.

It is unclear why the flexibility of the NUC domains increases upon dimerization. Do conformational changes in the HEL1/HEL2 domains result in different NUCHEL1/HEL2 interactions, thereby increasing NUC flexibility?

We appreciate this perspective from the reviewer and agree that additional clarification should be provided regarding the increased flexibility of the NUC domain upon Hna dimerization. We have provided more explicit depictions of domain rearrangements that occur upon Hna dimer formation (**Figure 2D**), with improved text to better explain the bilateral rearrangements of the Hna HEL2 domains that directly contribute to NUC domain flexibility via an unstructured linker:

"Critically, we observed that the NUC domain in protomer A was only partially resolved, while fully unresolved in protomer B, indicating a high degree of flexibility in this region (Fig. 2C). Rearrangement of a network of alpha helices in the HEL2 domain of protomer B promotes flexibility of the NUC domain via a long, unstructured linker (Fig. 2D). These asymmetrical movements provide a physical basis for the differential resolvability of the NUC domain in each Hna protomer and, consistent with our AF3 predictions, suggests that NUC flexibility may contribute to decreased nuclease capacity."

The authors should provide figures showing comparisons between the monomer and dimer (protomers A and B) regarding the overall structures and NUC-HEL1/HEL2 interfaces, as well as comparisons between protomer A and protomer B from the same angle (including superimposition if it is informative). Why is the NUC domain in protomer A partially disordered while that in protomer B is fully disordered in the dimer structure?

We agree with the reviewer that additional representations of Hna domain rearrangements upon dimerization would improve the structural characterization provided in this study. As mentioned above, we have improved our depictions of domain rearrangements upon dimer formation by directly superimposing our monomer and dimer structures (**Figure 2D**).

The most important question is why Hna exonuclease activity is inhibited upon dimerization (i.e., enhancement of the flexibility of the NUC domain), as the NUC active sites appear to be located outside rather than inside the dimer. This should be clarified.

We greatly appreciate this input from the reviewer. As previously mentioned, we more deliberately illustrate how global rearrangements contribute to increased NUC domain flexibility upon Hna dimer formation (**Figure 2D**). Furthermore, we have introduced new analysis accompanied by the development of an additional Hna mutant (PDE Hna) that is deficient in Mg^{2+} binding. We show that Hna exhibits distinct kinetic partitioning between its nuclease and ATPase active sites to moderate native levels of NUC activity (**Figure 3**). We speculate with the following text that the the NUC domain's greater dependency on metal ions may contribute to its enhanced flexibility and lack of activity in the dimeric state:

"While PD-(D/E)XK nucleases often employ a two metal ion mechanism of DNA cleavage, several instances of three metal ion coordination have been reported within this family and provide additional structural support necessary for catalysis[62, 63, 64]. We speculate that the increased sensitivity of Hna nuclease activity on metal ion concentrations may be attributed to additional ion coordination

requirements. Domain rearrangements upon ATP binding and subsequent dimer formation may also disrupt or weaken metal ion binding within the NUC active site, providing an explanation for the lack of exonuclease activity and increased flexibility of the NUC domain in our Hna dimer structure.”

> Does the helicase domain participate in ssDNA binding? AF3-predicted models of the Hna monomer and dimer bound to ssDNA might provide additional insights into the recognition and cleavage of ssDNA substrates.

We agree with the reviewer that AF3 models would enhance our understanding of how Hna interrogates its DNA substrates in the absence of DNA-bound cryo-EM structures. To address this concern, we have provided AF3 models that predict the Hna DNA-binding domains as well as the DNA-binding footprint of the protein (**Extended Figure 2D**). Additionally, we have performed additional DNA cleavage experiments to validate the predicted single-stranded DNA binding footprint (**Figure 1G**).

“***Inspection of the AF3 model shows that the 9-nt substrate fully occupies the DNA binding site, likely recapitulating the DNA footprint of Hna. Additionally, overlay of the AF3 model with our determined Hna structure revealed a unique conformation of the NUC domain when bound to the 9-nt ssDNA that appears largely disengaged from the HEL domains as observed in our Hna monomer structure. We speculate that the precise occupancy of the DNA binding domain results in non-productive rearrangements of the NUC domain, contributing to the notable loss of nuclease activity.”***

4. Fig. 1A-C: ATP concentrations should be specified in the legend, as they significantly affect the nuclease activity of Hna.

We thank the reviewer for pointing this out. The experiments referenced in this comment did not include ATP, however, we state this explicitly to avoid confusion:

“Hna exonuclease activity using fluorescently labeled, single-stranded DNA oligonucleotide in the presence of various metal ions and absence of ATP”

5. Figs. 2A and 3A: How can the authors determine if the shifted bands correspond to the Hna dimer rather than higher-order oligomeric states, even though the cryoEM structure indicates that Hna forms a dimer in the presence of ATP? It would be better to examine oligomeric states using other techniques, such as SEC, in addition to EMSA.

We agree with the reviewer that more analytical characterization of the basis of Hna dimerization would strengthen the claims in this study. To address this limitation, we performed a series of fluorescence anisotropy experiments using GFP-labeled Hna and showed that Hna oligomerizes readily in the presence of ATP. We also assessed our dimerization-deficient mutant and confirmed disruption of Hna dimerization (**Figure 2B**).

6. Fig. 3C: It would be informative to show the positions of phage escape mutations in the structure.

We appreciate this feedback. Although the positions of the phage escape mutations are colored uniquely in the current manuscript, we recognize that they are difficult to discern. To correct this issue, we have modified our color scheme and label these residues for improved clarity (**Figure 4A**).

7. Fig. 3H: Since these data are important to support the authors' model, gel data should also be presented either in the main or supplementary figure. Since Extended Data Fig. 3D (ATP dependency on Hna exonuclease activity) is important to support the authors' claim, it should be moved to Fig. 1.

We thank the reviewer for this suggestion. Due to the importance of showing SSB-mediated enhancement of Hna nuclease activity, we have performed additional gel-based cleavage experiments to complement the cleavage data obtained via quantitative capillarity electrophoresis (**Extended Figure 4C**). Additionally, we have moved Extended Figure 3D to a main figure as requested (**Figure 1H**).

Minor points:

1. Fig. 1A, B, etc.: A DNA size marker should be run alongside the samples and clearly labeled.

While we acknowledge that DNA size markers may provide additional clarity to the figures referenced by the reviewer, we believe that the inclusion of a protein-free DNA control is sufficient to show degradation of the substrate. Additionally, we include experiments using capillary electrophoresis of the same DNA substrate to accurately index the size of the DNA products produced by Hna (**Figure 1G, Extended Figure 1C**).

2. Fig. 1F, H, etc.: It would be beneficial to revise the color scheme to differentiate between the various conditions.

We greatly appreciate this input from the reviewer. We have revised our color scheme to provide greater contrast and clarity between conditions tested throughout the manuscript (**Figure 3A**).

3. Fig. 2B: The two bound ADP molecules should be labeled, or an explanation should be included in the legend. Additionally, it would be helpful to indicate the likely location of the disordered NUC domain in protomer B with a dashed circle.

We agree with the reviewer that the dimeric structure could be more clearly annotated. Due to overestimation of the resolution of the initial Hna dimer map, we have removed the bound ADP molecules from this model. Additionally, we have incorporated dashed circles to indicate the flexible portions of the NUC domains in this state, as requested (**Figure 2C**).

4. Extended Data Figs. 3B, C, and 4: Some labels are too small (less than 5 pt) to be discernible. These figures should be revised to enhance readability.

We thank the reviewer for this insight. We have increased the text size of all labels to improve readability of these data (**Extended Figure 5**).

REVIEWER #2

1. The mechanistic model is entirely in vitro and untested in vivo. Although prior work showed that 5A SSB can trigger Hna-dependent abortive infection, this manuscript does not test whether any of the newly proposed mechanistic steps occur in cells. Specifically, ATP-dependent autoinhibitory dimerisation, SSB-driven dimer disruption, and the behaviour of escape mutants are never validated in a biological system. There are no infection assays, toxicity assays, or cellular readouts using the mutants they generated. This leaves a major gap between the biochemical mechanism and physiological relevance.

We agree with the reviewer that the inclusion of in vivo experiments would improve the validity of the claims presented in this study. While we do maintain that the seminal work on the Hna system (Sather et al., 2023) provided extensive in vivo characterization, we aim to address this issue by performing a series of cellular toxicity assays for each of our Hna mutants. More specifically, we demonstrate that expression of Hna mutants with dysregulated nuclease activity result in enhanced toxicity, consistent with the requirement for tight regulation of nuclease activity under native conditions (**Figures 3H, 3I**).

2. The central Hna–SSB interaction is not rigorously demonstrated. The heterodimer model is built predominantly from AlphaFold3 predictions. Direct biochemical proof of interaction is limited to a qualitative native gel showing a shifted species. The single-molecule DNA-curtain experiments ,which should be the clearest assay , show almost no consistent colocalisation between GFP–SSB and Hna. Without quantitative binding assays (ITC, BLI, MST) or clear single molecule co-detection(fluorescence microscopy or even a pulldown), the central conclusion that SSB physically disrupts the Hna dimer remains under-supported.

We appreciate the insight provided by the reviewer and agree that more quantitative binding experiments would enhance our understanding of these interactions. To assess this, we performed a series of fluorescence anisotropy experiments using either GFP-tagged Hna or GFP-tagged SSB. We demonstrated that Hna and the 5A SSB readily form a higher-order species in a concentration-dependent manner. Additionally, the wild-type 5A SSB, and not the escape mutants, are sufficient to disrupt the autoinhibited Hna homodimer (**Figures 4B, 4C, 4F**).

"Given that Hna likely exists as an autoinhibited dimer under physiological conditions, we questioned whether introduction of the 5A SSB was sufficient to destabilize this dimeric state. To assess this, we performed additional fluorescence anisotropy experiments using saturating levels of GFP-Hna homodimer complex. We then titrated unlabeled 5A SSB and observed a significant decrease in anisotropy in the presence of ATP, suggesting the phage-encoded SSB promotes dissolution of the autoinhibited Hna dimer (Fig. 4F)."

3. No DNA-bound Hna structure, leaving substrate-binding and catalytic engagement speculative. The authors never observe DNA density in their cryo-EM structures. Yet the model depends on precise hypotheses about DNA engagement, transient contacts, and NUC-domain positioning. Without structural evidence for even a minimal DNA footprint, the mechanistic interpretation, and the claim that Hna has a "shallow" binding mode, remains speculative. While I understand the difficulties of the technique, it'd be better of the speculative nature of some of the conclusions was more clearly stated.

We agree with the reviewer that the lack of a DNA-bound structure weakens our understanding of how Hna interrogates its DNA targets. To address this limitation, we have performed a series of AF3 predictions accompanied with additional DNA cleavage experiments to estimate the DNA footprint of Hna. We hope that the introduction of AF3 prediction models and additional biochemistry will offer an improved perspective on Hna target DNA engagement (**Figure 1G, Extended Figure 2D**).

4. The nuclease domain is unresolved in the dimer, but the inhibition model depends entirely on its displacement. Large portions of the NUC domain are missing from the EM density in the dimer structure, yet the authors attribute ATP-dependent inhibition to NUC-domain misalignment and flexibility. This is inferred, not shown. Without a resolved NUC domain conformation in the inhibited dimer, the structural basis of inhibition is incomplete. Again, I recognise the EM challenges, but a more clearly stated "limitation of the study" or speculative nature of some of the conclusions would be appreciated.

We appreciate the perspective provided by the reviewer and agree that we do not show a step-by-step mechanism of Hna autoinhibition in the current manuscript. We have minimized the claims in our results text to avoid this interpretation of the work. To address this limitation experimentally, we have also generated an additional Hna mutant that is deficient in NUC domain metal ion binding (PDE Hna). We have introduced additional DNA cleavage and ATPase data using this Hna mutant to demonstrate that Hna exhibits distinct kinetic partitioning to regulate NUC activity under native conditions (**Figure 3**). Additionally, we speculate with the following text that the the NUC domain's greater dependency on metal ions may contribute to its enhanced flexibility and lack of activity in the dimeric state:

"While PD-(D/E)XK nucleases often employ a two metal ion mechanism of DNA cleavage, several instances of three metal ion coordination have been reported within this family and provide additional structural support necessary for catalysis[62, 63, 64]. We speculate that the increased sensitivity of Hna nuclease activity on metal ion concentrations may be attributed to additional ion coordination requirements. Domain rearrangements upon ATP binding and subsequent dimer formation may also disrupt or weaken metal ion binding within the NUC active site, providing an explanation for the lack of exonuclease activity and increased flexibility of the NUC domain in our Hna dimer structure."

5. Escape mutant analysis is not mechanistically convincing. The authors observe higher-order oligomers or higher DNA affinity in some mutants and infer this explains escape. But they never test:
– whether these mutants fail to bind Hna,
– whether they fail to disrupt the Hna dimer,
– whether they fail to stimulate nuclease activity,
– or whether the same effects occur in vivo. The mechanistic interpretation is therefore correlative.

We agree with the reviewer that more in-depth characterization of the SSB escape mutants would enhance the interpretation of the study. To further characterize Hna-SSB heterodimer formation, we performed a series of fluorescence anisotropy experiments using GFP-tagged Hna and non-fluorescent SSB and observed that the escape mutants largely fail to disrupt the autoinhibited Hna homodimer (**Figure 4F**). Additionally, we would like to acknowledge that the current manuscript does demonstrate that the SSB mutants fail to stimulate Hna nuclease activity (**Figures 4G, 4H**). Lastly, we believe in vivo characterization of this interaction is beyond the scope of the current work; however, we aim to provide a more comprehensive mechanistic framework for future studies.

6. Several results that anchor major conclusions (EMSAs, gel shifts, nuclease gels) are qualitative. Band intensities, ratios of monomer/dimer, and kinetics of nuclease activity are not quantified. For a mechanistic paper, this weakens the robustness of the claims.

We believe that the current manuscript provides extensive quantification of enzyme function including DNA cleavage activity, ATPase activity, and DNA-binding affinities (**Figures 1H, 1I, 2B, 2F, 3G, 4I**); however, we have also provided additional observed rate constants and binding affinities for all relevant DNA cleavage and binding experiments, respectively.

7. ATP inhibition mechanism is underdeveloped. While the authors show ATP inhibits nuclease activity, they do not evaluate:
   – ATPase kinetics under physiological ATP/ADP ratios,
   – the equilibrium between monomer and dimer across ATP concentrations,
   – whether ATP hydrolysis meaningfully contributes to dimer dissolution,
   – or whether SSB influences ATPase dynamics. Thus, the inhibitory mechanism lacks depth.

We appreciate the reviewers input and agree that the ATPase functionality of Hna could be more thoroughly explored. We have performed fluorescence anisotropy experiments using GFP-tagged Hna to show ATP-dependent Hna dimer formation (**Figure 2B**). Additionally, we show that Hna uses kinetic partitioning, mediated largely through ATP binding, to regulate nuclease activity under physiological conditions (**Figure 3**). Lastly, we show that none of the 5A SSB variants meaningfully influence Hna ATPase function (**Extended Figure 4D**).

8. Ambiguity in single-molecule conclusions. While the DNA-curtain experiments confirm that Hna binds and diffuses on ssDNA, they do not reveal any SSB-dependent changes in Hna behaviour. Hna does not show increased binding, altered diffusion, or enhanced processivity upon addition of 5A SSB. More importantly, co-localisation between Hna and SSB on ssDNA is extremely rare, meaning the experiments never directly visualise the proposed SSB–Hna heterodimer or any SSB-triggered dissociation of the autoinhibited Hna dimer. As a result, the single-molecule observations fall short of demonstrating the central mechanistic claim that SSB physically engages and activates Hna.

We agree with the reviewer’s comments and acknowledge that this relationship between Hna and the SSB is accommodated by the proposed mechanism in the current manuscript (**Figure 5**). The DNA curtains experiments, accompanied with our native gel shift assays suggest that Hna and the SSB interact independent of or prior to DNA binding. We have introduced additional, DNA-independent fluorescence anisotropy experiments to show that Hna and the SSB interact independent of DNA (**Figure 4C**).

9. The Discussion exaggerates the novelty of SSB-triggered activation. The text repeatedly frames this mechanism as unusual, although similar SSB dependent activation exists in Hachiman, AbpAB, Nhi, and others. A more measured comparison to known precedents would improve accuracy and framing.

We thank the reviewer for their insight on this issue. We have corrected this issue by more appropriately relaying the novelty and impact of this work:

“Hna functions as a single-effector ssDNA exonuclease with highly-conserved SF2 helicase domains involved in ATP hydrolysis and dimerization. We describe modes of Hna auto-inhibition and activation by a phage-encoded SSB. This mechanism illustrates how ATP-dependent oligomerization and metal-

sensitive catalytic partitioning can be integrated into a tunable immune switch. More broadly, our findings expand the functional repertoire of SF2 helicase-associated nucleases and highlight kinetic partitioning as a versatile strategy for balancing immune activation with host viability. Future work is needed to resolve discrete modes of DNA-binding and catalysis by Hna. We anticipate that continued investigation of Hna orthologs will reveal diversity in function and recognition of phage-encoded factors across members of the Hna family of bacterial immune systems."

Minor concerns:

1. Reporting of single-molecule assay conditions is incomplete or inconsistent. Key experimental parameters (protein concentration, injection concentration, flow rate, dwell times, surface density, and imaging buffer composition) are scattered through the Methods. This makes it difficult to assess reproducibility or the sensitivity of the observations.

We appreciate the input and agree that the description of the single-molecule DNA curtains experiments could be improved. We have added the detailed single-molecule experimental process with key parameters in the Methods section:

"The mixture was then diluted to a total volume of 100 µL imaging buffer with 200 nM final protein injection concentration. Immediately after the conjugation and dilution, the fluorescently labeled protein, Hna-ATTO647, was injected into the flowcell at a 0.12 mL min-1 flow rate. During image acquisition, the exposure time was set to 60 ms, with an interval of 1–2 seconds between frames. To observe Hna diffusion events, flow was temporarily turned off during imaging.

To image the binding of Hna on ssDNA and its colocalization with 5A SSB-GFP, 100 µL volume of Hna-ATTO647 protein (final concentration 200 nM) in ATP imaging buffer was injected into the flowcell, followed by injection of SSB-GFP (final concentration 1 nM) in ATP imaging buffer using 10 mL syringe for at least 10 minutes.The buffer flow was set to 0.2 mL min-1 flow rate. During image acquisition, the exposure time was set to 60 ms, with an interval of 2 seconds between frames."

2. ATP concentrations differ across experiments without rationale or discussion. Some assays use 20 µM ATP (ATPase assays), others use 1 mM ATP (nuclease assays, EMSAs, DNA curtains). The physiological relevance of these concentrations is not addressed, nor is it explained why different ATP levels are used for different readouts

We acknowledge that ATP concentrations differ slightly throughout the manuscript. For all relevant cleavage or binding experiments we use 1 mM ATP, and we have added clarification to reflect the physiological significance of this concentration. The use of a lower concentration of ATP for the ATPase assays is due to the limit of detection for the assay.

"Additionally, substitution of ATP for the non-hydrolyzable analog, ADP·BeF3, yielded comparable inhibition of DNA degradation, suggesting that Hna enters an inhibited state upon ATP binding rather than following ATP hydrolysis (Extended Fig. 3C)[43, 44, 45]. These data suggest that ATP acts as a negative regulator of Hna nuclease activity with considerable sensitivity at even sub-physiological levels of ATP (<1 mM)[46, 47]."

3. Figure presentation needs clearer lane and marker annotations. Several gel-based figures (especially native gels and cleavage assays) lack molecular weight markers, clear sample labels, or schematics of which lanes contain which components. This makes interpretation harder than necessary.

While we acknowledge that DNA size markers may provide additional clarity to the figures referenced by the reviewer, we believe that the inclusion of a protein-free DNA control is sufficient to show degradation of the substrate. Additionally, we believe that the gels presented in the current manuscript are sufficiently labeled to illustrate the contents of each reaction.

4. No quantitative or structural analysis of NUC-domain flexibility. The nuclease domain is unresolved in the dimer structure, yet the inhibition model relies on its displacement. The manuscript provides no quantification (e.g., local resolution, variance maps) to support claims about its flexibility

We would like to acknowledge that the current manuscript does provide quantification in the form of local resolution maps (**Extended Figure 5**) that reflect the decreased resolution and flexibility of the NUC domains in the dimeric structure.

5. The comparison between monomer and dimer cryo-EM maps is insufficiently explicit. While the text mentions conformational rearrangements, the figures do not include direct overlays or domain-specific RMSD comparisons that would clarify how ATPbinding drives inhibition.

We appreciate this perspective from the reviewer and have provided more explicit depictions of domain rearrangements that occur upon Hna dimer formation (**Figure 2D**), with direct superimpositions of the monomer and dimer structures and improved text to better explain the bilateral rearrangements of the Hna domains that directly contribute to NUC domain flexibility via an unstructured linker:

"Critically, we observed that the NUC domain in protomer A was only partially resolved, while fully unresolved in protomer B, indicating a high degree of flexibility in this region (Fig. 2C). Rearrangement of a network of alpha helices in the HEL2 domain of protomer B promotes flexibility of the NUC domain via a long, unstructured linker (Fig. 2D). These asymmetrical movements provide a physical basis for the differential resolvability of the NUC domain in each Hna protomer and, consistent with our AF3 predictions, suggests that NUC flexibility may contribute to decreased nuclease capacity."

REVIEWER #3

The manuscript "Phage-encoded factor stimulates DNA degradation by the Hna anti-phage defense system" submitted by Hooper et al. uses a combination of biophysical and biochemical measurements to describe the molecular mechanism of the Hna anti-phage immune system encoded by S. meliloti. My role as a reviewer is limited to assessment of the technical aspects of the cryo-EM work so I will comment only on that. Unfortunately, I do not find the maps of the Hna complex very compelling – visual inspection suggests the resolutions are substantially worse than the reported 3.3 Å for the dimer and 3.4 Å for the monomer. Indeed, the PDB validation reports (which should have been submitted with the manuscript for review) estimate the unmasked

resolutions to be 4.49 Å (dimer) and 3.95 Å (monomer), which look more appropriate. Furthermore, the maps appear to be aggressively sharpened and the sharpening process is not described in the materials and methods.

We appreciate the reviewer's in-depth analysis of the cryo-EM structures presented in the current study. We shared a folder with the unsharpened and sharpened reconstructions and the half-maps with our initial submission. It's unclear why that information wasn't transmitted properly. Regardless, please see this link with the updated data: https://utexas.box.com/s/85rqpdxh469z2v2i5vuiiitioh8um6yh. We agree with the reviewer that the previously reported resolution of the dimer structure reported from cryoSPARC was overestimated. This may have led to the observation of aggressive sharpening. However, we did not employ aggressive or additional sharpening tools. The structures are the output of non-uniform refinement within cryoSPARC. To address these issues, we have followed the suggestions from the reviewer and more appropriately reported the resolution of our monomer and dimer structures as 3.9 Å and 4.4 Å, respectively (**Figures 1D, 2C**).

Due to the low resolution of the maps, the protein models are also not convincing except as general footprints showing the relative placement of protein domains. Rigid body fitting of domains from Alphafold3 predictions is appropriate into this type of map, but I am not convinced that refinements with Coot, Isolde, and Phenix are meaningful. Especially worrisome is the placement of ligands like calcium ions and ADP – these are not supported by the data. The poor map-model fits are reflected in the low atom inclusion scores and Q-scores present in the PDB validation reports. Overall, this cryo-EM work is not up to par for a key piece of data in this manuscript. If the manuscript were re-written to honestly communicate the limitations of the maps and models I would reconsider. But as written, the manuscript is misleading as to the quality of the structural biology work and I must therefore decline to recommend it for publication. I know high resolution maps are very challenging to achieve for small molecules, and I commend the authors for what they were able to accomplish – but I believe there must be more transparency in the description of the resulting data.

As requested, we have modified our models to omit all bound ligands from both the monomeric and dimeric structures. Additionally, we have limited our interpretation of these maps and models to general observations regarding domain placement and rearrangements. We have removed all side chains from our dimer model, and focused our interpretations based on rigid-body fitting domains from the monomer and interpretation of more global changes in Hna conformation. We have now prioritized the biochemical findings in this study and reference the cryo-EM structures with an emphasis on AF3 predictions to support our observations (**Figures 1D, 2C**).

"Despite limitations in the final resolution of this structure, we were able to rigid-body fit domains of each Hna protomer to visualize notable domain rearrangements upon Hna dimerization. Hna assembles into a homodimer with psuedo-C2 symmetry via a highly conserved, unstructured loop in the HEL1 domain (Fig. 2C, 2E). This interaction is stabilized through an asymmetric shift of the HEL1 domain of protomer A, increasing availability of the dimerization interface (Fig. 2D). Critically, we observed that the NUC domain in protomer A was only partially resolved, while fully unresolved in protomer B, indicating a high degree of flexibility in this region (Fig. 2C). Rearrangement of a network of alpha helices in the HEL2 domain of protomer B promotes flexibility of the NUC domain via a long, unstructured linker (Fig. 2D). These asymmetrical movements provide a physical basis for the differential resolvability of the NUC domain in each Hna protomer and, consistent with our AF3 predictions, suggests that NUC flexibility may contribute to decreased nuclease capacity."
